# Activation of Wnt Signaling by Chemically Induced Dimerization of LRP5 Disrupts Cellular Homeostasis

**DOI:** 10.1371/journal.pone.0030814

**Published:** 2012-01-27

**Authors:** Payam Shahi, Dongsu Park, Adam C. Pond, Mamatha Seethammagari, Shin-Heng Chiou, Kyucheol Cho, Julienne L. Carstens, William K. Decker, Pierre D. McCrea, Michael M. Ittmann, Jeffrey M. Rosen, David M. Spencer

**Affiliations:** 1 Department of Pathology and Immunology, Baylor College of Medicine, Houston, Texas, United States of America; 2 Department of Molecular and Cellular Biology, Baylor College of Medicine, Houston, Texas, United States of America; 3 Department of Biochemistry and Molecular Biology, University of Texas MD Anderson Cancer Center, Houston, Texas, United States of America; The National Institute of Diabetes and Digestive and Kidney Diseases, United States of America

## Abstract

Wnt signaling is crucial for a variety of biological processes, including body axis formation, planar polarity, stem cell maintenance and cellular differentiation. Therefore, targeted manipulation of Wnt signaling *in vivo* would be extremely useful. By applying chemical inducer of dimerization (CID) technology, we were able to modify the Wnt co-receptor, low-density lipoprotein (LDL)-receptor-related protein 5 (LRP5), to generate the synthetic ligand inducible Wnt switch, iLRP5. We show that iLRP5 oligomerization results in its localization to disheveled-containing punctate structures and sequestration of scaffold protein Axin, leading to robust β-catenin-mediated signaling. Moreover, we identify a novel LRP5 cytoplasmic domain critical for its intracellular localization and casein kinase 1-dependent β-catenin signaling. Finally, by utilizing iLRP5 as a Wnt signaling switch, we generated the Ubiquitous Activator of β-catenin (Ubi-Cat) transgenic mouse line. The Ubi-Cat line allows for nearly ubiquitous expression of iLRP5 under control of the H-2K^b^ promoter. Activation of iLRP5 in isolated prostate basal epithelial stem cells resulted in expansion of p63^+^ cells and development of hyperplasia in reconstituted murine prostate grafts. Independently, iLRP5 induction in adult prostate stroma enhanced prostate tissue regeneration. Moreover, induction of iLRP5 in male Ubi-Cat mice resulted in prostate tumor progression over several months from prostate hyperplasia to adenocarcinoma. We also investigated iLRP5 activation in Ubi-Cat-derived mammary cells, observing that prolonged activation results in mammary tumor formation. Thus, in two distinct experimental mouse models, activation of iLRP5 results in disruption of tissue homeostasis, demonstrating the utility of iLRP5 as a novel research tool for determining the outcome of Wnt activation in a precise spatially and temporally determined fashion.

## Introduction

Wnt signaling exerts diverse biological effects in an organism, such as body axis determination during embryogenesis, cell polarity induction and stem cell self-renewal [1]. Mutations that result in the over-activation of the Wnt signaling axis have been identified in many cancers and shown to play critical roles in tumorigenesis [2], [3]. Due to the central importance of Wnt signaling in development and maintenance of tissue homeostasis, molecular interactions involved with Wnt pathway induction and its final physiological outcome have been widely investigated. To date, there are 19 known Wnt ligands and 10 different frizzled (Fz) family Wnt receptors identified in mammals. Additionally, there are several Wnt co-receptors, such as lipoprotein receptor-related protein (LRP)-5/6, the axon guidance protein, Receptor related to tyrosine kinase (Ryk) and ROR1/2 [4], [5], [6]. The combinatorial interactions of these diverse proteins likely result in a vast array of biological effects. Wnt signaling may be propagated through the canonical Wnt/β-catenin pathway or via numerous “non-canonical” signaling pathways, affecting disparate targets, such as planar cell polarity, Jun kinase (JNK) activation or intracellular calcium levels.

Association of Wnt ligand to its receptor/co-receptor complex initiates canonical Wnt signaling. In the absence of Wnt ligands, the β-catenin “destruction complex” comprising Axin, adenomatosis polyposis coli (APC) and GSK-3β, sequesters and phosphorylates the canonical Wnt signal transducer, β-catenin. Phosphorylated β-catenin is targeted for ubiquitination and proteasomal degradation. Hence, in the unliganded state, the cytoplasmic pool of β-catenin is maintained at low levels. Upon association of a Wnt ligand to an appropriate Fz receptor and co-receptor LRP5/6, the regulatory protein Dishevelled (Dvl) becomes activated via phosphorylation. Activated Dvl triggers disruption of the destruction complex, allowing for nuclear accumulation of β-catenin, where it interacts with the LEF/TCF complex, resulting in the transcription of Wnt target genes.

Due mostly to lipid modifications, purification of Wnt proteins has proven to be technically challenging. Therefore, in order to reproduce Wnt activation in experimental settings, various strategies have been developed to selectively stabilize or upregulate β-catenin expression. Although *in vitro* experiments have many practical benefits, such as typically shorter duration and high throughput data analysis, the complexity of multi-lineage systems attainable in genetically engineered mouse (GEM) models often provides a more physiologically relevant understanding of a particular biological process. Since the technique was developed in the early 1980s, many transgenic mouse models have been characterized. Manipulation of regulatory pathways, via selective gene regulation in transgenic models, has provided important insights into tumor development and cancer biology. More recent “conditional” technologies, such as Cre-Lox recombination or tamoxifen-inducible transcriptional regulation, have added temporal control of *in vivo* gene manipulation. These systems have proven to be especially useful in models where alteration of a biological pathway may result in embryonic lethality. However, reversible regulation of gene expression or protein function can provide an additional dimension of control to permanent Cre-Lox-induced changes.

The importance of Wnt signaling in tumor biology has led to a keen interest in dissecting and identifying key regulatory components. The mouse mammary tumor virus (MMTV)-based tumor model provided initial insight into the importance of Wnt signaling in the regulation of tissue homeostasis. Int-1 (Wnt1) was the first gene discovered to be activated by an integrated provirus in the MMTV tumor models [7]. Following the discovery of Wnt1, numerous Wnt pathway-targeted knockout mouse models have been developed in order to decipher the molecular details of Wnt signaling [8]. Moreover, by taking advantage of the Cre-Lox system, multiple mouse models have been engineered that also permit spatiotemporal control of Wnt signaling activation. One such method utilized the conditional inactivation of APC to simulate Wnt signaling activation. Conditional deletion of exon 14 in the APC^580S/580S^ mouse model (APC^flox/flox^) promoted adenoma formation in the colorectal region by 4 weeks of age [9]. In a separate study, prostate-targeted, probasin-Cre^+^ (PB-Cre^+^) mice were crossed with APC^flox/flox^ to generate PB-Cre^+^; APC^flox/flox^. These mice allow for the inactivation of APC specifically within prostate epithelial cells, leading to development of prostate adenocarcinoma within 7 months [10], again consistent with a key role for β-catenin in tumor progression.

A second method for conditional activation of Wnt signaling involves stabilization of β-catenin by excising a portion of exon 3, the target of phosphorylation and subsequent ubiquitination and degradation. Transgenic mice expressing conditionally activated β-catenin in neural precursors develop enlarged brains and increased cerebral cortical surface area [11]. In prostate epithelial-targeted β-catenin activation models, prostate tumor formation occurs, although the kinetics is slower than in the APC KO model [12], [13]. Together, these mouse models have expanded our knowledge of aberrant Wnt signaling activity in various tissues.

Considering the prevalence of Wnt signaling-based mouse models that are constitutively active, we opted to develop an inducible canonical Wnt signaling system that utilizes the more versatile chemical inducer of dimerization (CID)-based “on/off” system. Toward this goal, we performed CID-based oligomerization of several components of Wnt signaling and discovered that inducible multimerization of the cytoplasmic domains of the Wnt coreceptors, LRP5 and LRP6, could emulate canonical Wnt signaling, without the potentially confounding effects of LRP5/6 interaction with Wnt inhibitors, Dickkopf proteins (DKKs) and sclerostin (SOST) [14], [15]. Inducible LRP5 (iLRP5), comprises the cytoplasmic portion of LRP5 fused to the CID-binding domain, FKBP12(V36) (F_v_). Like a “true” switch, iLRP5 has the ability to be turned on and off by the simple addition or removal of CID, AP20187, resulting in potent canonical Wnt pathway induction.

To better understand the disparate outcomes of ß-catenin induction in physiological settings, we used the broadly active major histocompatibility complex I (MHC-I) promoter (H-2K^b^) to express iLRP5 and a coupled click beetle (*species*) red-shifted luciferase reporter (CBR) gene. This allowed for the near ubiquitous expression of iLRP5, as well as bioluminescence imaging in live tissues. Initially, we have analyzed the consequence of Wnt activation via iLRP5 dimerization in mammary and prostate tissue systems. Both of these systems exhibited highly reproducible CID-dependent hyperplasia and in some instances tumorigenesis. These transgenic mice, named Ubiquitous Activator of β-catenin (or “Ubi-Cat”), should, therefore, provide a valuable research tool for controlled induction of the Wnt pathway in various tissues.

## Results

### Synthetic recruitment of LRP5/6 to lipid rafts leads to inducible β-catenin regulation

**Figure 1:**
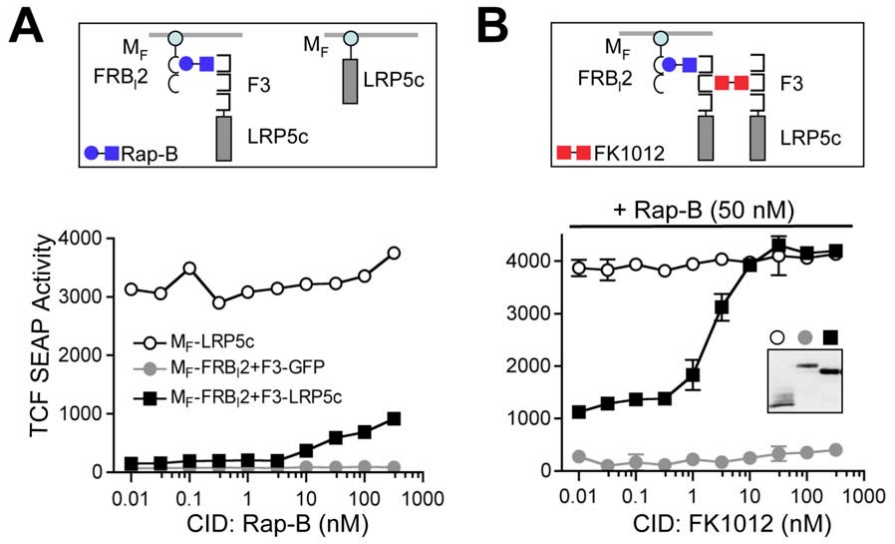
LRP5 forms constitutive dimers and CID-mediated dimerization of LRP5 induces TCF/β-catenin activity. Schematics (top) and TCF reporter activity (bottom) of CID-mediated membrane translocation (**A**) with dimerization (**B**) of the LRP5 cytoplasmic domain (LRP5c). 293 cells were transiently transfected with TOP-SEAP reporter plasmid along with plasmids encoding myristoylated LRP5c (M_F_-LRP5c) or lipid raft-docking construct, M_F_-FRB_l_2, along with either LRP5c fused to 3 tandem rapalog-binding domains (F3-LRP5c) or control protein, F3-eGFP. TOP-SEAP activity was measured 24 hours after administration of indicated amounts of Rapalog-B (Rap-B) (**A**) or homodimer (FK1012) along with suboptimal levels of Rap-B (50 nM) (**B**). Protein expression levels are indicated by anti-HA western blot (inset).

Consistent with plasma membrane-anchored LRP5/LRP6 variants, lipid raft targeting of the intracellular domains of LRP5 and LRP6 (M_F_-LRP5c and M_F_-LRP6c) constitutively activates β-catenin signaling in mammalian cells, suggesting that membrane localization of the signaling domain of LRP5/LRP6 is a major determinant for β-catenin induction [16], [17], [18], [19](**Fig. S1**). Therefore, we reasoned inducible recruitment of the cytoplasmic domain of LRP5/LRP6 to membrane lipid rafts might result in reversible regulation of signaling. To test this, we fused the LRP5 cytoplasmic domain to three tandem rapamycin-binding FKBP12(P89, K90) domains [20], generating F3-LRP5c. As a lipid raft-docking protein, we fused the myristoylation-targeting domain of c-Fyn (M_F_) to tandem copies of the mutant, 89-amino acid, rapamycin-binding domain from mTOR (mammalian target of rapamycin; FRB(L2098)/FRB_l_) [21]. Upon administration of membrane-permeable rapamycin analogs (“rapalogs”) [20] to cells co-expressing F3-LRP5c and M_F_-FRB_l_2, β-catenin signaling is induced, as determined using the TCF4/β-catenin-responsive reporter, TCF-SEAP (**Fig. 1**, **left panels**). However, the signaling level was lower than permanently lipid raft-“anchored” LRP5 (M_F_-LRP5c), suggesting that membrane translocation alone is not sufficient for maximal β-catenin induction.

During lipid raft targeting, the high local concentration of proteins that occurs likely leads to increased incidental protein aggregation that could contribute to activation of signaling. Therefore we tested the hypothesis that further regulated oligomerization of membrane-targeted F3-LRP5c might further enhance β-catenin activation (**Fig. 1** **, top right**). Indeed, in addition to low-level β-catenin induction achieved by membrane localization, further dimerization (or oligomerization) of LRP5 cytoplasmic domains, via dimeric FK506, FK1012 [22], resulted in an increased level of β-catenin signaling (**Fig. 1** **, bottom right**), suggesting that synthetic dimerization of LRP5 recapitulates the reported functional role of LRP5/6 clustering and activation [23].

### Intracellular dimerization of LRP5c is sufficient to induce canonical Wnt signaling in an Axin-dependent manner

**Figure 2:**
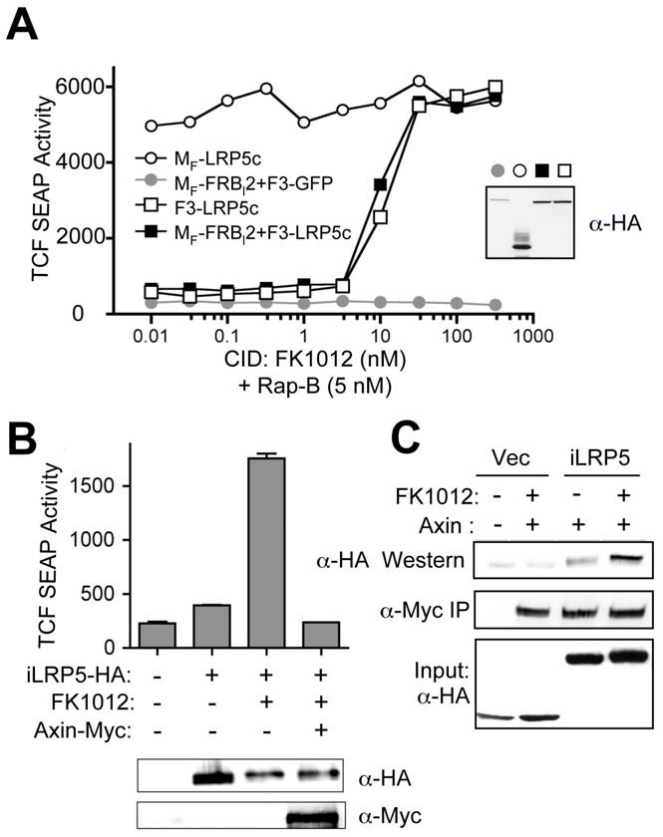
Axin is a critical downstream regulator of iLRP5 and interacts with iLRP5. (**A**) Effects of dimerization and membrane translocation of LRP5c on Wnt/β-catenin signaling. Activation of the TCF reporter in 293 cells transiently transfected with 1 µg each TCF-SEAP reporter and M_F_-LRP5c (○), M_F_-FRB_l_2+F3-GFP (•), F3-LRP5c (□) or M_F_-FRB_l_2+F3-LRP5c (▪) expression plasmids is shown. After 24 hours, cell aliquots were treated with CID and suboptimal (5 nM) Rap-B, and SEAP activity was measured after 24 hours. (**B**) Effect of Axin on iLRP5-mediated β-catenin activation. 293 cells were transiently transfected with 1 µg each of TCF-SEAP and iLRP5 plasmids with our without myc-tagged Axin. After 24 hours, cells were treated with 100 nM CID (AP20187) or diluent alone, and SEAP activity was measured after an additional 24 hours. Protein expression levels were determined by anti-myc and anti-HA blotting (inset). (**C**) Dimerization-dependent interaction of Axin and iLRP5. 293 cells were transfected with 1 µg of indicated combinations of iLRP5 and Axin. After treatment with 100 nM homodimer (AP20187) for 24 hours (lane 2, 4), whole cell lysates were immunoprecipitated with anti-myc antibody (myc). Co-immunoprecipitation of LRP5c was analyzed by anti-HA blot (HA). Backbone (F_v′_-F_vls_-HA) or iLRP5 (F_v′_-F_vls_-iLRP5-HA) protein expression was detected by anti-HA blot (Input: HA). All data are representative of at least two (panel A) or three (panels B–C) independent experiments with similar results. Error bars (B) represent S.D. of triplicate measurements.

Several reports have shown that LRP5 phosphorylation by membrane-associated kinases, such as CK1γ and GSK3β, is required for Axin sequestration and induction of canonical Wnt signaling, again suggesting membrane targeting of LRP5 is critical [24], [25]. Therefore, to determine if the observed dimerization-dependent LRP5c signaling requires membrane localization, we dimerized non-targeted LRP5c. Surprisingly, we observed that administration of FK1012 to cells transfected with F3-LRP5c alone (without docking protein M_F_-FRB_l_2), led to robust β-catenin induction, indistinguishable to that achieved by dimerization (or oligomerization) of membrane-localized F3-LRP5c (**Fig. 2A**, open square). This suggested the possibility that LRP5 oligomerization is sufficient for β-catenin stabilization and that membrane-localization may be necessary only to facilitate ligand-receptor interactions. To further assess this observation, we redesigned iLRP5 using the “second generation” CID-binding domain, FKBP12(V36) (“F_v_”), that responds to FKBP12v36 (F_v_)-selective CID, AP20187 (or clinical candidate AP1903) [26]. Consistently, simple multimerization of the cytoplasmic signaling domain of LRP5 led to potent induction of β-catenin activation, regardless of the lack of a classical membrane-targeting sequence (data not shown).

It has been well documented that sequestration of Axin from the destruction complex by membrane-bounded LRP5 is a key step for β-catenin induction (16). Therefore, we tested whether interaction with Axin by dimerized iLRP5 is required for β-catenin activation. Co-expression of Axin with M_F_-LRP5c or iLRP5 completely abrogated both M_F_-LRP5c- and iLRP5-mediated β-catenin activation, respectively (**Fig. 2B**, **S2**). In addition, dimerization of cytoplasmic iLRP5 induced the interaction of dimerized iLRP5 and Axin, suggesting that Axin is a critical downstream target of endogenous LRP5, as well as iLRP5 (**Fig. 2C**).

### Dimerized LRP5 co-localizes with Dvl but induces β-catenin in a Dvl and Axin-dependent manner

**Figure 3:**
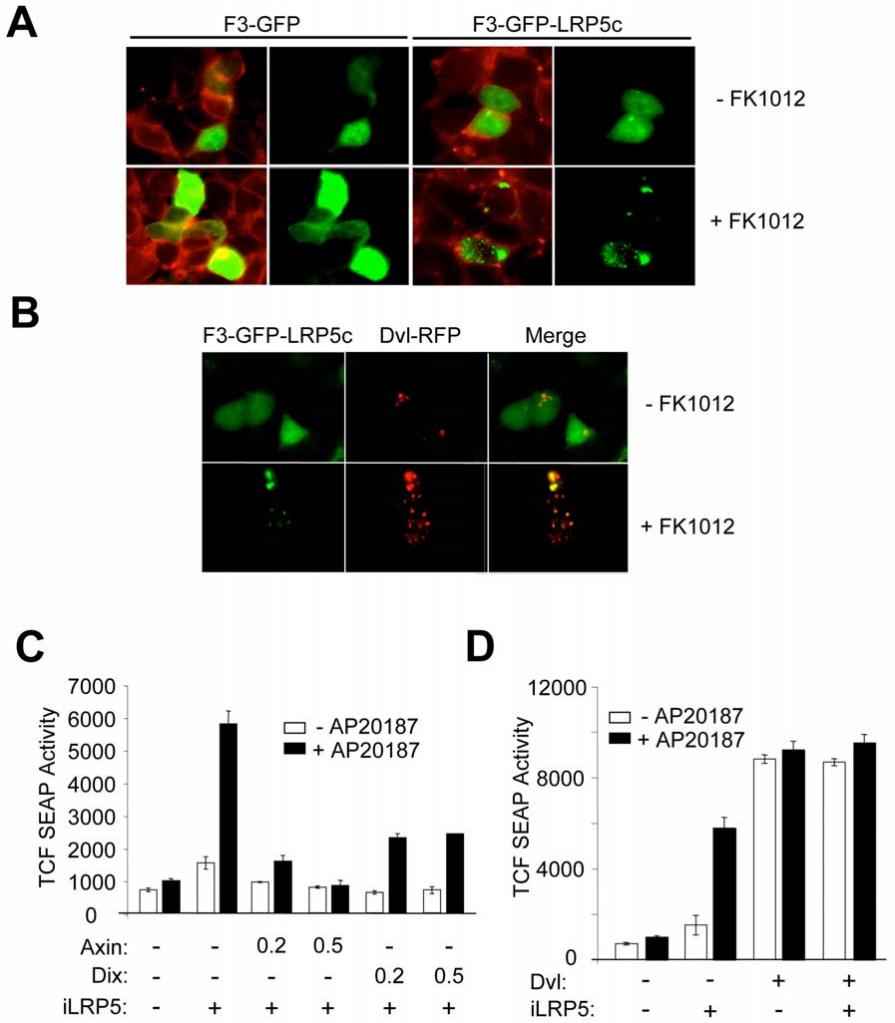
Dimerized-iLRP5 localizes to disheveled-containing punctate structures. (**A, B**) Subcellular localization of dimerized LRP5c and disheveled. 293 cells were transfected with 1 µg each of plasmids expressing drug-binding domains fused with GFP (F3-GFP) or GFP-LRP5c (F3-GFP-LRP5c) with (B) or without (A) RFP-fused disheveled construct, Dvl-RFP. Twenty-four hours after transfection, 100 nM FK1012 was added for an additional 24 hours to induce F3-LRP5c dimerization. After fixation, cells were labeled with cholera toxin B (CTx-B-TRITC) (A) and examined by fluorescence microscopy. (**C, D**) Independent role of iLRP5 and disheveled in β-catenin activation. 293 cells were transfected with 1 µg TCF-SEAP reporter along with 0.5 µg of iLRP5 and the indicated amounts (µg) of Axin or dominant negative disheveled (Dix) constructs (C) or 0.5 µg disheveled construct (D). Twenty-four hours after transfection, 100 nM CID was added and SEAP activity was measured after 24 hours. Data is representative of at least three independent experiments (A, B). Error bars indicate mean ± S.D. of triplicate measurements (C. D).

Although dimerized F3-LRP5c could stabilize β-catenin without direct membrane localization, we could not rule out that dimerization of LRP5c had conferred increased membrane affinity, possibly due to Fz/Dvl complexes, consistent with the putative role for Dvl in LRP6 aggregation in the plasma membrane [23]. Rather than increasing plasma membrane localization, however, we found that dimerization of F3-GFP-LRP5c (containing an eGFP domain to permit direct visualization) induced a punctate, cytoplasmic pattern, suggesting that dimerized LRP5c might associate with intra-cytoplasmic membrane compartments, possibly containing the Fz/Dvl receptor complex (**Fig. 3A**). Further studies showed that these iLRP5-containing punctate structures did not overlap with endosomes or lysosomes (data not shown), but instead co-localized with Dvl-containing punctate structures in the cytoplasm (**Fig. 3B**). To demonstrate this, we fused red fluorescence protein (RFP) to full-length Dvl to produce Dvl-RFP and examined subcellular expression with or without F3-GFP-LRP5c. Interestingly, Dvl-RFP specifically co-localized with F3-GFP-LRP5c only after dimerization of LRP5c, but not in control cells, suggesting that active and dimerized LRP5c translocates to unique Dvl-containing punctate structures.

Although Dvl has been reported to associate with Frizzled receptors (Fz) during Fz-mediated Wnt signaling, it remains unresolved whether iLRP5-mediated β-catenin signaling is also Dvl-dependent. Previous findings have revealed the importance of Dvl in mediating LRP6-signalosome assembly [23]. Therefore, we were interested in a possible role of Dvl in iLRP5-mediated Wnt signaling activation. Therefore, we tested whether a dominant-negative Dvl allele, comprising primarily the Axin-binding, N-terminal DIX domain of Dvl, could affect iLRP5-mediated β-catenin activation. Although 0.2 µg of Axin expression vector almost completely blocked iLRP5 activity, 0.5 µg of DIX expression vector showed only a partial inhibition of iLRP5-mediated β-catenin activation (**Fig. 3C**). In addition, co-expression of iLRP5 and Dvl showed moderately additive effects on β-catenin activation, suggesting that although iLRP5 translocates to intracellular Dvl-containing punctate structures, iLRP5-mediated Axin-β-catenin signaling may still involve Dvl as part of the iLRP5 signalosome (**Fig. 3D**). Finally, in order to ensure that the above observations are not due to inconsistencies in DNA uptake during transfection, protein expression for individual assays was examined via Western blot analysis **(Fig. S3)**.

### CK1 is a critical downstream regulator of iLRP5-mediated β-catenin activation

**Figure 4:**
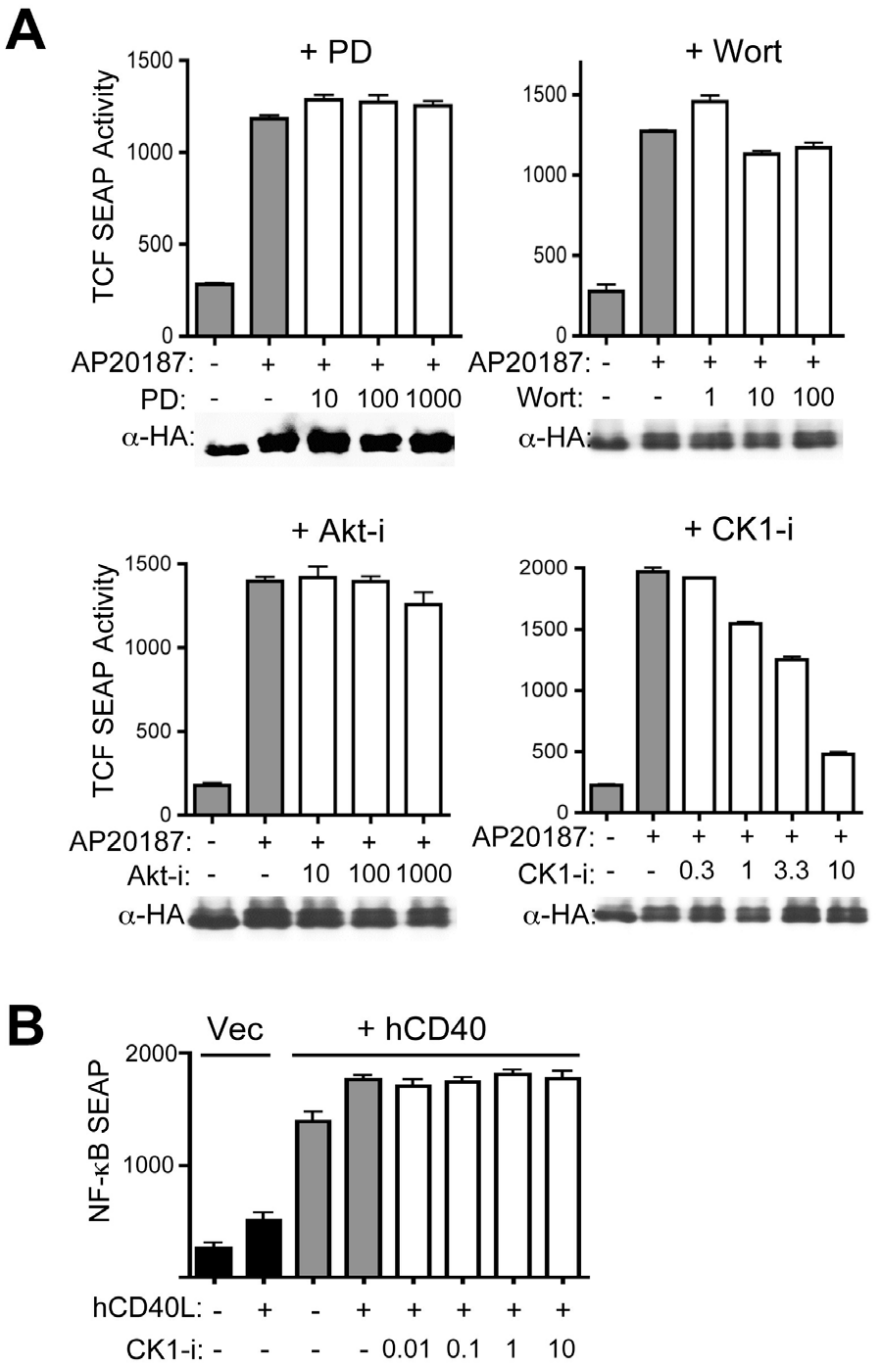
CK1 is required for iLRP5-mediated β-catenin activation. (**A**) 293 cells were co-transfected with 1 µg TCF-reporter and iLRP5 construct and incubated for 24 hours with indicated concentrations (nM) of MAPK (PD98059 (PD)), PI3K (wortmannin (Wort)), Akt (Akt-i), or casein kinase 1 (CK1-i) inhibitor. (**B**) 293 cells were co-transfected with 1 µg NF-κB-reporter and hCD40 construct and incubated for 24 hours with indicated nM concentrations of CK1-i plus hCD40L (1 µg/mL). SEAP activity was measured after an additional 24-hour incubation with or without 100 nM CID (AP20187). Data is representative of at least two independent experiments. The amount of protein expression was normalized by anti-HA blot (α-HA).

Previous reports suggested LRP5 activation is regulated by phosphorylation of its cytoplasmic domain by membrane-associated CK1γ. [24], [25]. However, we observed that CID-activated iLRP5 is localized in the cytoplasm yet still highly functional. In order to test whether CK1γ is also required for iLRP5-mediated β-catenin activation, we first tested the effects of CK1 inhibition using CK1-i. Although p42/44 MAPK, PI3K and Akt inhibitors (i.e. PD98059, wortmannin, and Akt-i, respectively) did not inhibit CID-dependent iLRP5 activity, the CK1 inhibitor, CK1-i, clearly blocked iLRP5-mediated β-catenin activation in a dose-dependent fashion. Similar CK1-i levels had no effect on hCD40/CD40L-mediated NF-κB activation (**Fig. 4**). These results support that both dimerization of LRP5c and CK1 activity are required for iLRP5-mediated β-catenin activation.

### Both a novel region (1516–1531) of LRP5 and CK1 phosphorylation sites are critical for iLRP5 function and subcellular localization

**Figure 5:**
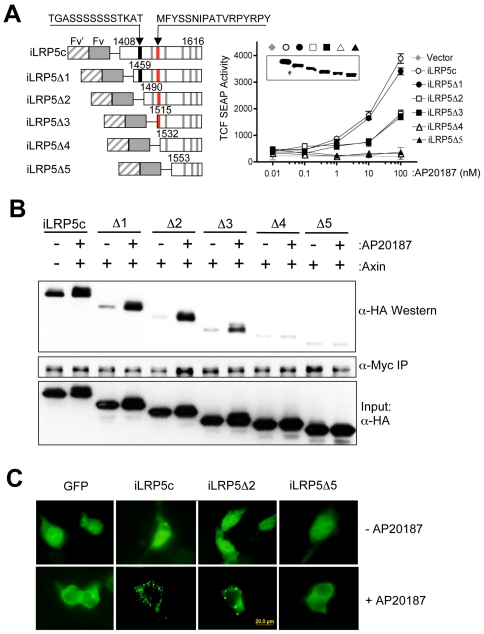
Two distinct regulatory sites in LRP5c are critical for function and localization of iLRP5. (**A**) (left) Schematic of LRP5c deletion mutants (iLRP5 Δ1–5) comprised of synthetic ligand-binding domains (F_v′_-F_vls_). Numbers indicate N-terminal amino acid position of each deletion mutant. Red and black lines represent indicated sequences and gray lines represent PPPSP motifs, respectively [24]. (right) Domain analysis of LRP5c on Wnt/β-catenin signaling. 293 cells were transiently transfected with 1 µg each of indicated constructs along with TCF-SEAP reporter. After 24 hours, cell aliquots were treated with indicated concentrations of AP20187, and SEAP activity was measured after an additional 24-hour incubation. Data represent two independent experiments with similar results. (**B**) Role of novel and CK1 sites in the interaction of Axin and iLRP5. 293 cells were transfected with 1 µg each of indicated mutants of iLRP5 along with an Axin expression construct. After 24-hour treatment with 100 nM AP20187 (+CID), Axin was immunoprecipitated from whole cell lysates with anti-myc antibody (α-myc). Co-immunoprecipitation of LRP5c was analyzed by anti-HA blot (α-HA). Amount of iLRP5 mutant expression was determined by HA blot (Input: α-HA). (**C**) Subcellular localization of iLRP5-truncation mutants. 293 cells were transfected with 1 µg of indicated plasmids. Twenty-four hours after transfection, 100 nM FK1012 was added for an additional 24 hours to induce dimerization. After fixation, cells were examined by fluorescence microscopy.

It has been proposed that plasma membrane-associated CK1γ phosphorylates two Ser/Thr-containing motifs in LRP6 [24]. Therefore, to further define the potential role of CK1 phosphorylation sites in iLRP5, corresponding to CK1-targeted sites in LRP6, we designed a deletion series of iLRP5 constructs and tested them for β-catenin-inducing ability. These included full-length iLRP5c (LRP5_1408–1615_) and iLRP5Δ1 (LRP5_1456–1615_) containing two potential CK1 sites, iLRP5Δ2 (LRP5_1490–1615_) containing one CK1 site, and iLRP5Δ3 (LRP5_1515–1615_), iLRP5Δ4 (LRP5_1532–1615_) and iLRP5Δ5 (LRP5_1553–1615_) containing only “PPPSP” consensus sites without CK1 sites. Interestingly, we observed that the deletion of just one CK1 site in iLRP5 dramatically reduced β-catenin activation (compare iLRP5Δ1 and Δ2; **Fig. 5A, B**). However, the deletion of a second CK1 site found in iLRPΔ2 [24] did not further affect iLRP5-mediated Wnt/β-catenin signals, showing indistinguishable activity compared with iLRP5Δ3 (**Fig. 5A**). Instead, the further deletion of a previously unidentified region of LRP5 (_1516_MFYSSNIPATVRPYRPY_1531_) in constructs iLRP5Δ4 and iLRP5Δ5, which do not carry any consensus CK1 phosphorylation sites [24], completely abolished residual Wnt/β-catenin activity, suggesting that the phosphorylation of iLRP5 by CK1 and additional factor(s) targeting adjacent regions are critical for iLRP5 function.

Subsequent co-immunoprecipitation studies between iLRP5 mutants and Axin revealed that the deletion of CK1 motifs and a domain (1516–1531) in iLRP5Δ4 and iLRP5Δ5, which contain at least three PPPSP motifs, also was correlative with downregulation of the Axin/iLRP5 interaction, suggesting that a possible defect of PPP(S/T)P phosphorylation in iLRP5 deletion mutants contributes to reducing Axin/iLRP5 interaction (**Fig. 5B**). Therefore, to further clarify the phosphorylation status of iLRP5 mutants after dimerization, we performed western analysis with anti-pS1490 antibody, which can specifically recognize the phosphorylated PPPSP motif, needed for Axin interaction (**Fig. S4**). Interestingly, both truncated LRP5-Δ2 and LRP5-Δ5 were constitutively phosphorylated even without dimerization, indicating that dimerization and CK1 phosphorylation rather than phosphorylation of PPP(S/T)P domains in LRP5 is required to induce iLRP5-mediated β-catenin activation.

To further examine the possible correlation of iLRP5c or iLRP5c mutants with their subcellular localization, GFP fusion proteins with LRP5c deletion mutants were generated. Consistently, iLRP5Δ1 dimerization induced the distinct punctate cytoplasmic structures (similar to those observed with iLRP5c), whereas dimerization of iLRP5Δ2 and iLRP5Δ5 showed progressive loss of subcellular localization (iLRP5Δ3 showed the same pattern as iLRP5Δ2), indicating that re-localization to punctate structures after dimerization of iLRP5 exhibited a strong correlation with subsequent β-catenin activation (**Fig. 5C**).

**Figure 6:**
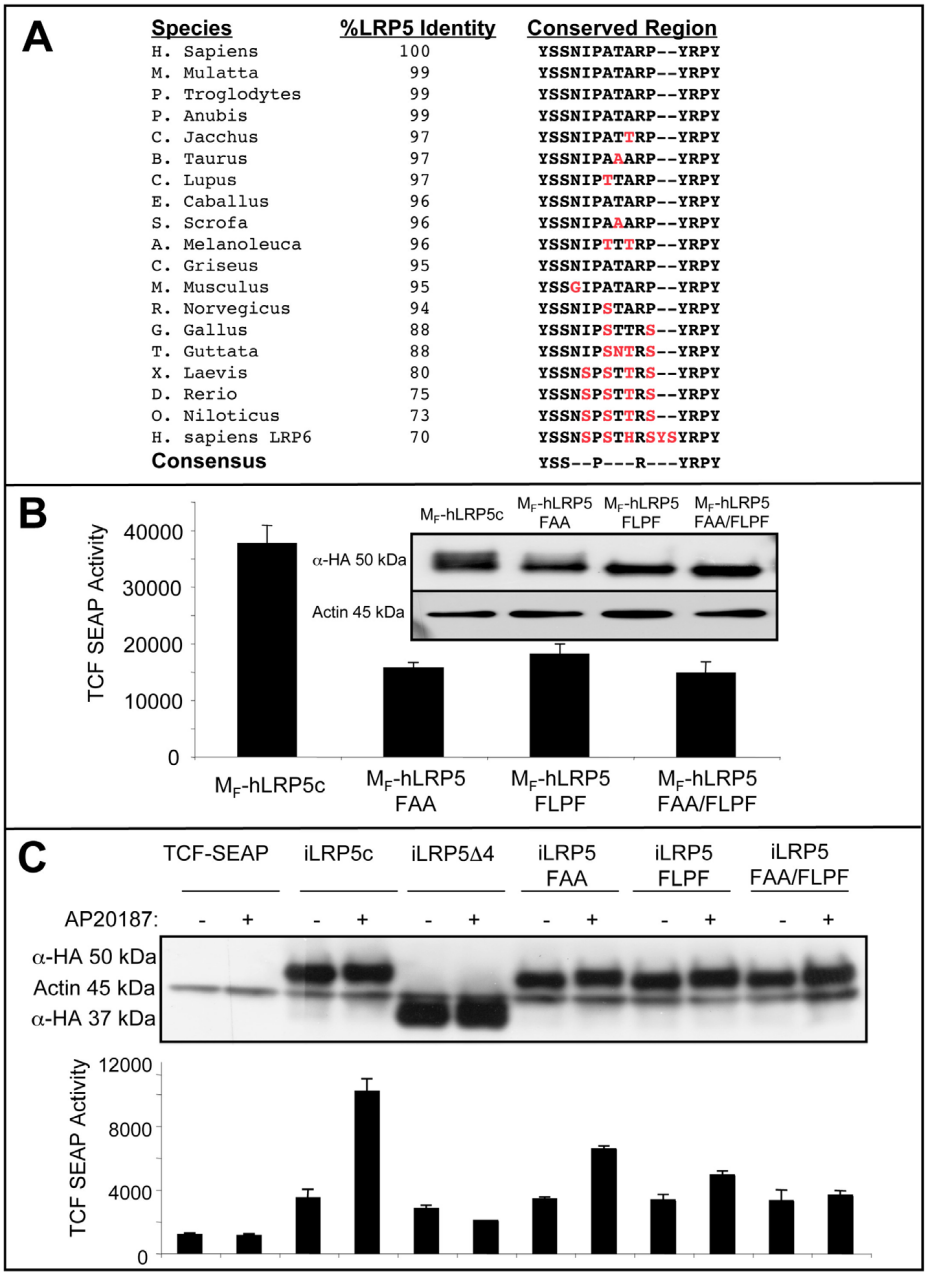
A novel region of LRP5 (_1516_MFYSSNIPATVRPYRPY_1531_) is required for Wnt signaling. (**A**) Cross-species analysis of the novel LRP5 (_1516_MFYSSNIPATVRPYRPY_1531_) sequence. (**B**) Membrane targeting of the cytoplasmic moiety of LRP5 (M_F_-LRP5) and three variants containing mutations of possible phosphorylation sites: (1) YSS→FAA, (2) YRPY→FLPF and (3) YSS→FAA and YRPY→FLPF. The mutants have reduced Wnt pathway inducibility. Western blot indicates comparable protein expression from respective TCF-SEAP assay. (**C**) Comparison of iLRP5 and iLRP5 mutants. YSS→FAA and YRPY→FLPF mutants have lower TCF-SEAP activity. The double mutant fails to activate Wnt signaling. Western blot indicates protein expression from respective TCF-SEAP assay. (B and C), Data representative of two separate experiments performed in triplicates.

The previously unidentified region of LRP5 (_1516_MFYSSNIPATVRPYRPY_1531_) appears to possess important residues required for full iLRP5/Wnt signaling. Sequence homology analysis of this LRP5-derived region from various species as well as a comparison to highly homologous and functionally related co-receptor LRP6 supports a critical role for several residues **(** **Fig. 6A** **)**. Interestingly, two highly conserved subregions within the sequence (YSS and YRPY) contain possible phosphorylation target sites, thus helping to target a limited mutagenesis study. Initially, we generated three membrane-targeted M_F_-hLRP5 variants, utilizing the lipid raft-targeting amino terminus of c-Fyn. In one, we replaced Y1518 with phenylalanine and adjacent serines with alanines [Y1518F; S1519A; S1520A]. In a second, YRPY sequence was altered to FLPF [Y1529F; R1530L; Y1531F] and the third clone possessed both FAA and FLPF mutant sequences. A TCF-reporter assay was used to measure constitutive canonical Wnt signaling of all 3 mutants relative to M_F_-hLRP5, containing the unmutated sequence **(** **Fig. 6B** **)**. Curiously, although both FAA and FLPF mutants revealed reduced activity, the double-cluster mutant, containing FAA and FLPF residues had similarly reduced activity, possibly due to the inclusion of colocalized, endogenous LRP5 (or 6) in signaling-competent multimers.

To further dissect the importance of YSS and YRPY sequences, we generated three iLRP5 variants carrying the previously described mutations. We reasoned that non-membrane-localized iLRP5 variants would be less likely to interact with endogenous sequence. Interestingly, FAA, FLPF and FAA/FLPF double mutants now demonstrated distinct levels of Wnt activation upon cytosolic dimerization **(** **Fig. 6C** **)**. Whereas, the FAA and, to a lower extent, FLPF mutants demonstrated reduced β-catenin inducibility, the double mutant variant was completely inactive upon dimerization **(** **Fig. 6C** **)**. Overall, the above observation emphasizes the importance of the previously unidentified LRP5 (_1516_MFYSSNIPATVRPYRPY_1531_) region for iLRP5 activity as well as for full activity of membrane-targeted M_F_-hLRP5, designed to approximate endogenous LRP5 function.

### Ubi-Cat Transgenic Mice

**Figure 7:**
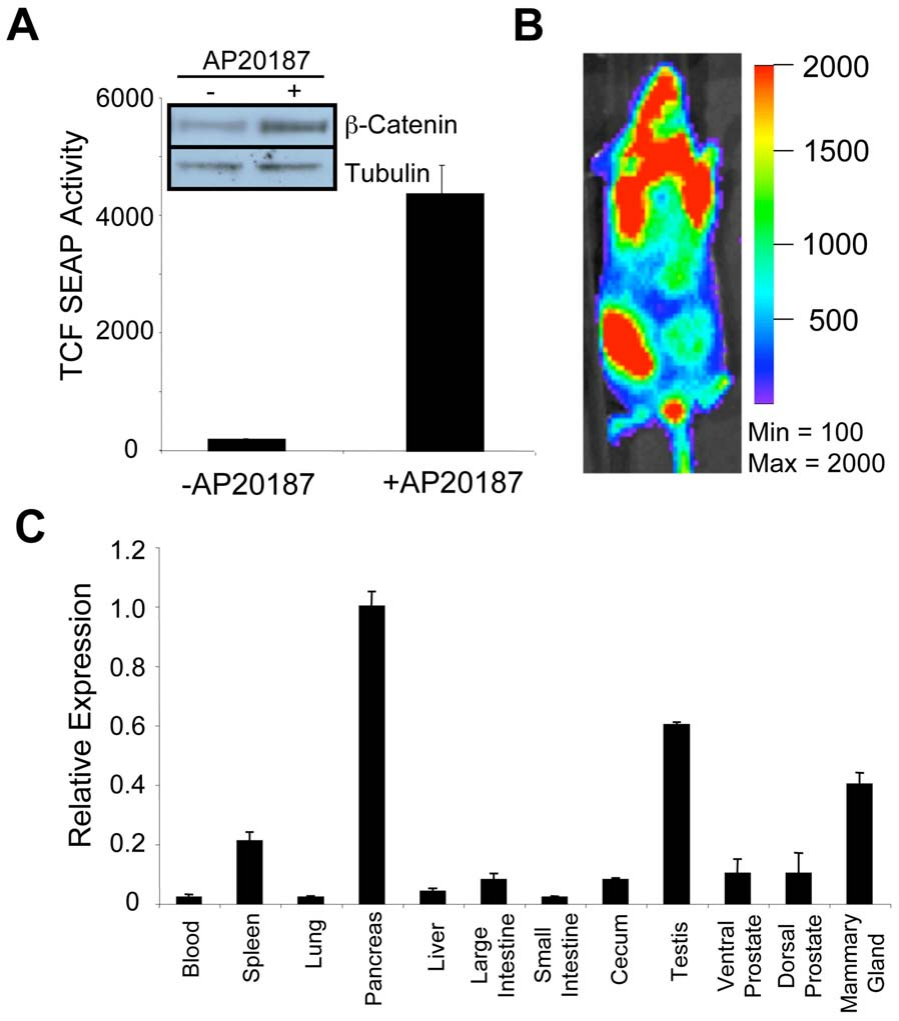
Analysis of iLRP5 expression in Ubi-Cat transgenic mice. (**A**) 293 cells were co-transfected with Ubi-Cat expression plasmid (0.5 µg) along with TCF-SEAP reporter (0.2 µg). Cells were induced with AP20187 (100 nM) for 48 hours. Cell lysate and media were collected for examination of iLRP5 activity. Western blot analysis indicates iLRP5-dependent stabilization of cytoplasmic β-catenin, consistent with accompanying SEAP reporter assay. (**B**). Analysis of CBR expression via IVIS. (**C**) qRT-PCR analysis of iLRP5 expression in selected Ubi-Cat tissues using a transgene-specific primer-probe set.

In order to investigate the physiological relevance of the iLRP5 switch as a Wnt signaling activator, we designed transgenic mice, termed “Ubiquitous expression of inducible β-Catenin (Ubi-Cat)”. To affect broad somatic cell expression of iLRP5, we utilized the H-2K^b^ (MHC- class I) promoter [27]. Initially, we examined Wnt signaling activation by the Ubi-Cat transgene in HEK-293 cells via a standard reporter assay. The Ubi-Cat construct showed titratable responsiveness to CID treatment and was able to induce Wnt reporter activity **(** **Figure 7A** **)**. CID-mediated iLRP5 dimerization also resulted in cytoplasmic stabilization of β-catenin **(** **Figure 7A** **)**.

Next we examined transgene expression in Ubi-Cat mice. The Ubi-Cat construct is coupled to a click beetle red-shifted luciferase (CBR) reporter, permitting transgene expression detection via *in vivo* bioluminescent imaging. Ubi-Cat mice demonstrated broad iLRP5 expression via IVIS imaging **(** **Fig. 7B** **)**. Moreover, iLRP5 expression via qRT-PCR was detected in multiple tissues of adult mice **(** **Figure 7C** **)**. Among the tissues examined, iLRP5 expression was highest in the pancreas, followed by testes, mammary gland, spleen and prostate.

### iLRP5 Induction Promotes the Expansion of Prostate p63^+^ Basal Epithelial Cells

One unique aspect of the Ubi-Cat mouse model is the putative expression of the iLRP5 switch in various cell types within tissues. Therefore, different cell types from a particular tissue could potentially be purified for further analysis following Wnt signaling induction. Analysis of Wnt signaling activation in fractionated cells versus intact tissue should provide useful information about cell autonomous versus non-cell autonomous influences in a particular tissue. Due to multiple reports of a strong correlation between increased Wnt signaling and prostate cancer [28], we initially analyzed the outcome of iLRP5-mediated Wnt pathway induction in purified prostate basal epithelial stem cells (B/SCs), using markers Lin^−^, Sca-1^+^, CD49f^+^ (providing the moniker, LSC cells [29]).

**Figure 8:**
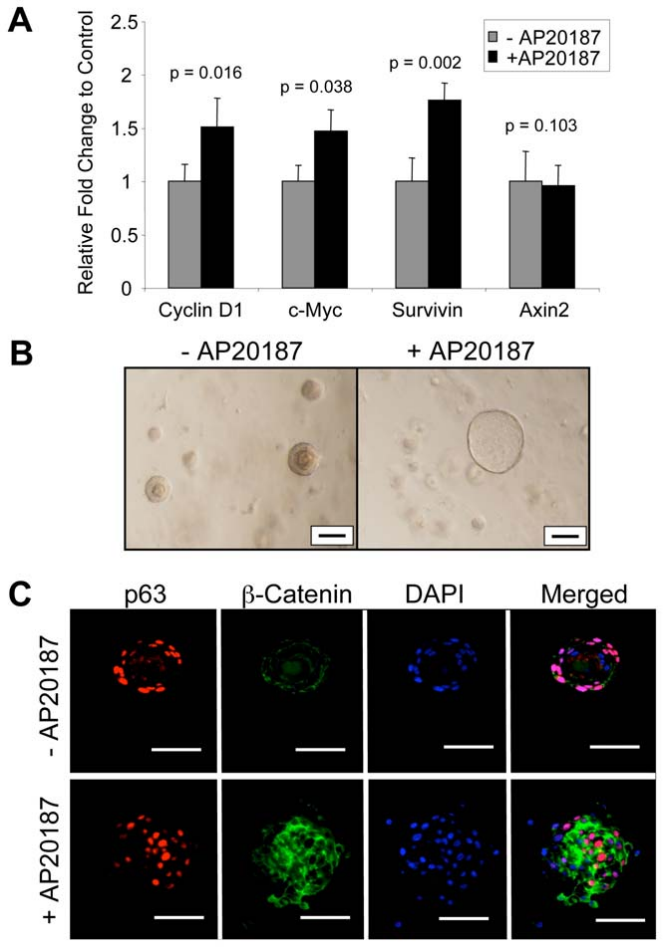
*In vitro* analysis of iLRP5 activation in prostate B/SCs. (**A**) qRT-PCR analysis of Wnt target genes. LSCs were cultured in Matrigel for 6 days with or without CID (100 nM). Cells were isolated and RNA was collected for cDNA synthesis and qRT-PCR analysis. CID-mediated iLRP5 activation promotes Wnt target genes expression. Error bars represent StDev of 3 experiments, normalized to 18S RNA (**B**) Prolonged (16 days) activation of iLRP5 promotes enlargement of prostaspheres. Control spheres grew to maximum size of 150–200 µm; however, CID-treated spheres grew to 300–400 µm. Bar = 100 µm. (**C**) Analysis of p63 and β-catenin expression in prostasphere cross-sections. iLRP5 activation enhances β-catenin stability. CID-treated spheres reveal dispersed distribution of p63^+^ cells within all layers of prostasphere. Bar = 100 µm.

In order to demonstrate that CID-mediated induction of iLRP5 in LSCs promotes the activation of canonical Wnt target genes, we performed qRT-PCR for known, canonical Wnt target genes, including cyclin D1, c-myc, survivin and Axin2. iLRP5 activation resulted in ∼1.5-fold increase in steady state RNA for cyclin D1 and c-myc, and ∼2-fold increase in survivin expression. However Axin2 RNA levels were unaffected **(** **Fig. 8A** **)**.

Next, we utilized LSCs from 6–8-week-old Ubi-Cat mice for *in vitro* prostasphere assays as a surrogate assay for stem cell self-renewal or *in vivo* prostate reconstitution experiments. Generally, the *in vitro* assays are performed in the extracellular matrix material, Matrigel™ seeded with 1×10^4^ LSCs. CID-mediated activation of iLRP5 resulted in consistent prostasphere enlargement **(** **Fig. 8B** **)**, which paralleled our previous data obtained by utilizing purified Wnt3a ligand [30]. Moreover, Wnt signaling activation resulted in higher β-catenin staining and dispersal of p63^+^ cells **(** **Fig. 8C** **)**. Cross-sectional analysis of iLRP5-induced spheres revealed disruption of cellular organization of p63^+^ cells. In untreated prostaspheres, p63^+^ cells generally reside on the outer most cell layer, while the inner layers comprise mostly compacted cells and are largely devoid of p63 expression. In contrast, iLRP activation results in the generation of spheres comprising dispersed p63^+^ cells within all cell layers and the lack of cellular compaction within the inner most layers. Thus, iLRP5-induced spheres appear to phenocopy our previous observations utilizing Wnt3a ligand [30], [31].

**Figure 9:**
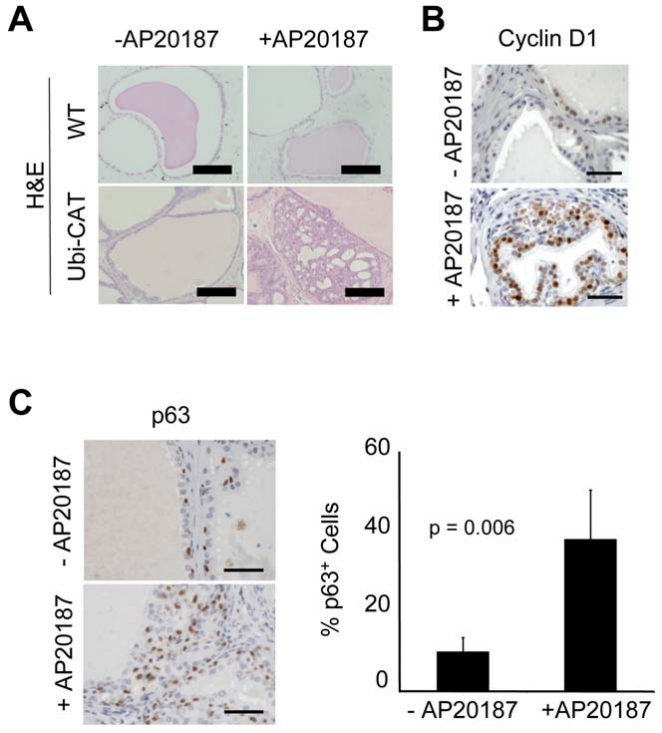
Activation of iLRP promotes prostate hyperplasia in the prostate reconstituted grafts. (**A**) H&E analysis of prostate grafts. Activation of iLRP5 for 8 weeks resulted in formation of hyperplastic acini in prostate grafts. Bar = 100 µm. (**B**) Immunostaining of prostate grafts using cyclin D1 antibody. Bar = 50 µm (**C**) Immunostaining of prostate grafts using p63 antibody, Activation of iLRP5 resulted in over two-fold increase in the number of p63^+^ cells. Data representative of 9 +CID and 9 −CID grafts. Bar = 50 µm.

In order to examine the cell-autonomous effects of iLRP5 activation in LSCs during prostate regeneration, we used a prostate reconstitution assay. Multiple sub-Q grafts, containing 1×10^5^ WT LSC or Ubi-Cat LSCs and 1×10^5^ WT E18 rat-derived urogenital sinus mesenchyme (UGSM) cells mixed in Matrigel, were transplanted into the backs of CD1*^nu/nu^* mice. The LSC grafts were analyzed 8 weeks after transplantation. In the WT control or Ubi-Cat-derived grafts in the absence of CID, glandular structures were generally composed of simple monolayer structures; however, hyperplasia was widespread in CID-treated grafts **(** **Fig. 9A** **)**. iLRP5 activation in LSC grafts also resulted in enhanced cyclin D1 staining, suggesting that iLRP5 activation increases cyclin D1 activity **(** **Fig. 9B** **)**. Analysis of p63 expression revealed that there was a two-fold increase in the number of p63^+^ cells in the iLRP5-induced, reconstituted grafts **(** **Fig. 9C** **)**. Again, *in vivo* observations based on iLRP5 activation in LSCs support previously described results relying upon constitutively active β-catenin [30], supporting the utility of iLRP5 as a Wnt-inducible switch in prostate B/SCs.

### Activation of iLRP5 in the Prostate Stroma Enhances Prostate Reconstitution

**Figure 10:**
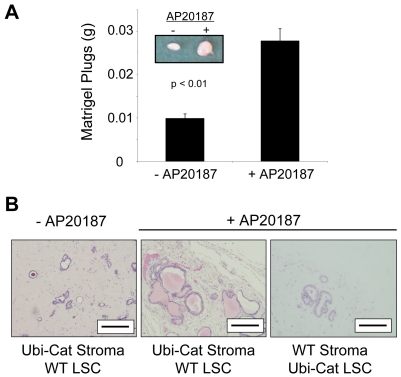
Analysis of prostate reconstitution comprising adult Ubi-Cat prostate stroma and WT LSCs. (**A**) Activation of iLRP5 increases size and weight of treated grafts. (**B**) Induction of iLRP5 in prostate stroma enhances prostate regeneration in treated grafts. Data representative of 6 +CID and 6 −CID grafts. Bar = 200 µm.

Prostate homeostasis is maintained via balanced communication between the stroma and the underlying epithelial compartment. Therefore, we were interested in the effects of iLRP5 induction in the prostate stroma. Since we have already established a working model for *in vivo* prostate regeneration, we thought that this model could potentially be useful to examine the effect of Ubi-Cat-derived stroma in a developing prostate graft. Sca-1^+^, CD49f^−^, Lin^−^ Ubi-Cat prostate stromal cells (1×10^5^) were mixed with WT LSCs (1×10^5^ cells) 1∶1 in Matrigel. The grafts were subsequently injected sub-Q into the backs of CD1*^nu/nu^* mice followed by 8 weeks of CID treatment. Visual inspection and weight measurement of the grafts indicated that the CID-treated samples were physically larger and heavier **(** **Fig. 10A** **)**. Cross-sectional analysis of the grafts demonstrated that in the absence of CID or in control reconstituted grafts using WT stoma and Ubi-Cat LSCs, prostate acini were underdeveloped and lacking in prostate secretions. However, in CID-treated grafts, the stroma was more densely populated with cells and prostate ducts developed to a normal size (∼200 µm) that contained histologically normal prostate secretions **(** **Fig. 10B** **)**. The observed phenotype suggests that iLRP5 is functional in Ubi-Cat stromal cells, as well as LSCs. Moreover, activation of Wnt signaling in prostate stroma via iLRP5 is sufficient to drive proper prostate development. These observations support an important role of Wnt signaling and its regulation during prostate development with possible cell autonomous functions in both basal LSCs and stromal cells.

### Induction of iLRP5 in Ubi-Cat Mice

To examine the outcome of iLRP5 activation in intact prostate tissue, a cohort of Ubi-Cat mice were treated bi-weekly with CID (AP20187, 50 µg/mouse) for various durations. Animal cohorts were treated starting at 6 weeks of age for 4, 8, 20, 36, 44, and 52 weeks and prostate were analyzed upon sacrifice. Despite low-level, but prolonged β-catenin induction, treated mice appeared healthy during the course of these studies. CID-mediated iLRP5 activation *in vivo* resulted in nuclear β-catenin localization by immunostaining **(Fig. S5)**. Activation of iLRP5 for up to 20 weeks further resulted in a modest hyperproliferation of prostate epithelial cells **(Data not shown)**. Whereas age-matched, untreated mice showed slight proliferation among ventral lobe epithelial cells, treated mice reflected higher proliferative rates in both ventral and the dorsal lobes. The epithelial cells developed luminal hyperplasia and the normal single-cell layer organization of luminal cells was replaced by hyperplasia. We also observed a modest stromal thickening, consistent with reactive stroma. Furthermore, the severity of the proliferative phenotype was gradually enhanced with increased CID treatment, but not apparent in age-matched, untreated mice.

**Figure 11:**
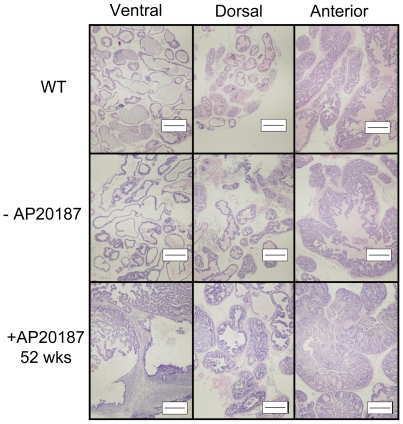
*In vivo* CID-mediated iLRP5 activation in the intact prostate results in prostate tumor development. Treatment of Ubi-Cat mice (n = 6) with AP20187 starting at 6 weeks of age for 52 weeks leads to development of prostate adenocarcinoma (2 of 6). Age-matched WT and untreated Ubi-Cat prostates (n = 6) appeared normal. Bar = 200 µm.

Treatment of Ubi-Cat mice beyond 20 weeks resulted in an even more prominent hyperproliferative phenotype in the ventral and the dorsal lobes. Cells in the ventral lobe began to fill the lumen and coincided with significant stromal thickening, as well as a moderate level of acinar disorganization. Some nuclei were hyperchromatic and atypical, appearing elliptical by H&E analysis. Similarly, the dorsal lobes also demonstrated significant epithelial proliferation and stromal thickening, which was so severe in some foci that the stroma appeared to completely engulf the epithelial compartment. Moreover, the majority of acini were filled-in with epithelial and/or stroma cells **(Fig. S6)**. After 52 weeks of CID treatment, two of six mice had developed prostate adenocarcinoma, particularly in the ventral lobes. The ventral lobe was extremely disorganized and the basement membrane was severely disrupted or even absent in some acini **(** **Figure 11** **)**. The dorsal and the anterior lobes were also affected. The epithelial cells had completely filled the lumens of the prostate ducts. In contrast, age-matched WT and untreated Ubi-Cat mice appeared relatively normal.

By utilizing the prostate gland as a model for analysis of Wnt signaling induction in Ubi-Cat mice, we confirmed iLRP5 as a useful inducible β-catenin switch. CID-dependent changes in the prostatic lobes, from mild epithelial proliferation to adenocarcinoma, supported the physiological relevance of iLRP5-mediated Wnt signaling activation.

### iLRP5 Wnt pathway induction drives mammary gland hyperplasia and tumor formation

**Figure 12:**
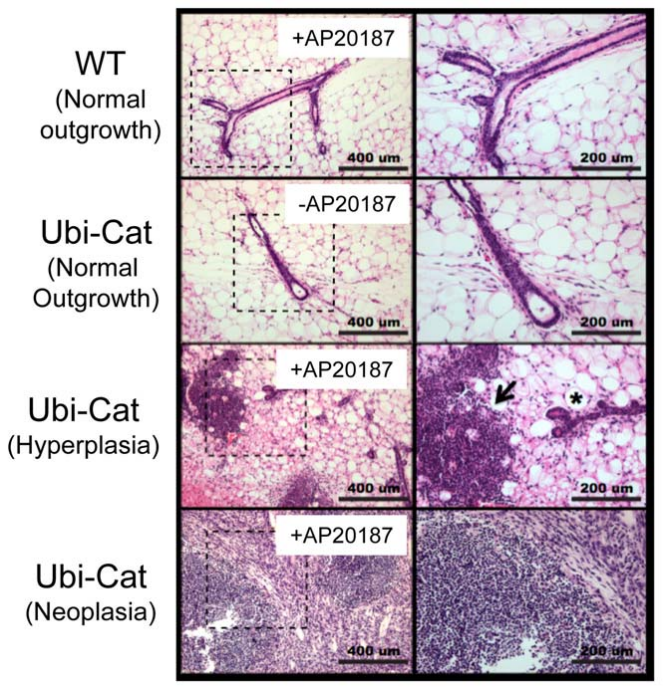
Mammary gland transplant assay using Ubi-Cat-derived mammary cells. Ubi-Cat mammary cells were isolated from 6–8 week adult female Ubi-Cat mice. 5×10^3^ WT or Ubi-Cat mammary cells were transplanted into virgin adult recipients. WT and untreated Ubi-Cat cells demonstrated normal mammary outgrowth 8 weeks after transplant. Activation of iLRP via AP20187 resulted in formation of hyperplastic mammary ducts in 4 of 6 grafts. Two of six CID-treated grafts formed mammary tumors.

To better appraise the broader utility of the Ubi-CAT model, we choose to examine the outcome of another tissue known to be influenced by Wnt signaling. Maintenance of cellular homeostasis in mammary epithelial cells is highly dependent on modulation of Wnt signaling. Based on existing data, there appears to be a direct correlation with deregulation of Wnt signaling and breast cancer development [7], [32]. In order to examine the physiological relevance and functionality of iLRP5 in Ubi-Cat mice, we explored the outcome of iLRP5-mediated Wnt signaling induction in transgenic mammary cells. Mammary epithelial cells from 8-week-old Ubi-Cat mice were transplanted into the fat pad of 3-week-old virgin female recipients. Each recipient was transplanted with 5×10^3^ transgenic cells on the left side and an equivalent number of WT cells on the right side. Transplanted mice were treated twice weekly with the AP20187 (CID) for 8-weeks prior to removal and analysis of grafted mammary cells. iLRP5-mediated Wnt pathway induction resulted in hyperplasia in 4 of 6 transplanted grafts, two of which developed mammary tumors **(** **Fig. 12** **)**. This was in comparison to normal mammary outgrowth observed in WT and untreated Ubi-Cat grafts. This mammary transplant data confirm that activation of canonical Wnt pathway signaling contributes to mammary gland hyperplasia and that Ubi-Cat transgenic mice can be used as a source of cells containing ligand-inducible ß-catenin expression.

## Discussion

Although it is widely accepted that the canonical Wnt/β-catenin signaling axis is initiated by Wnt-mediated Fz and LRP5/6 heterodimerization [33], [34], [35], key molecular details involved in canonical Wnt signaling propagation remain elusive, such as the details of how Dvl activates the dismantling of the destruction complex. Bilic et al. demonstrated that Dvl plays an integral role in clustering and phosphorylation of LRP6 and formation of the LRP6-signalosome. By utilizing real-time confocal microscopy they demonstrated that LRP6 aggregates in signaling foci within 15 min of Wnt stimulation, suggesting that oligomerization of LRP6 is a prerequisite for Wnt signaling propagation. The necessity for LRP6 clustering to promote Wnt signaling propagation provoked us to design an inducible Wnt system by marrying the LRP5/6 signaling domain with CID technology. In a previous study, Liu et al demonstrated that wild-type LRP6 also forms spontaneous, Wnt-independent dimers; however, these dimerized LRP6 subunits were inactive. Moreover, forced dimerization of the cytoplasmic LRP6 domain inhibited its activity [36], although, we also observed constitutive dimerization (or oligomerization) of LRP5c (data not shown). In contrast to that study, our results show that forced dimerization of LRP5c either at the plasma membrane or within the cytosol is sufficient to induce β-catenin stabilization (**Fig. 1**). Idiosyncrasies of the bulkier DNA gyrase B (30 kD)/coumermycin CID system used by Liu et al, relative to FKBP12 (12 kD)-based dimerization, could account for these disparate results. Consistent with our results, a separate study showed that dimerization of a membrane-associated LRP6 intracellular domain can stimulate signaling [37]. In addition, it has been reported that Frizzled receptors can also dimerize following Wnt binding, contributing to Wnt/β-catenin induction [38], [39]. Taken together, rather than inhibiting LRP5/6 signaling, these data support the conclusion that oligomerization of LRP5 and other Wnt-associated proteins can potentiate Wnt signaling.

Our study also reveals novel plasma membrane-distal aspects of LRP5 function. It has been generally accepted that plasma membrane localization of LRP5 precedes phosphorylation by membrane-associated CK1α, which is required for Axin sequestration and β-catenin stabilization [35]. However, using non-localized signaling domains and membrane-permeable ligands, we unexpectedly discovered that dimerization of cytosolic LRP5 is sufficient for localization to Dvl-containing punctate structures, Axin-binding and canonical Wnt signaling. This indicates a link between LRP5 oligomerization and recruitment to intra-cytoplasmic compartments that contain the Dvl/Axin complex. It is also tempting to speculate that a similar mechanism can explain the requirement for intracellular trafficking to an endosomal protein complex by the Wingless (Wnt homologue) signaling complex in *Drosophila melanogaster* that includes Arrow (LRP5 homologue) and Dishevelled [40].

Amino-terminal deletion and site-directed mutagenesis analysis of the cytoplasmic signaling domain of LRP5 revealed that a previously unidentified region of LRP5 (_1516_MFYSSNIPATVRPYRPY_1531_), devoid of consensus CK1 phosphorylation sites, is essential for β-catenin induction, and intra-cytoplasmic localization. Nonetheless, we consistently observed that CK1 activity is critical for LRP5 function. Since dimerization of iLRP5 within intra-cytoplasmic compartments appears functionally intact, it is likely that cytoplasmic CK1 isoforms, associated with Dvl/Axin complexes, are able to induce LRP5 phosphorylation and activation, comparable to membrane-bound CK1. However, it remains to be tested what additional factor(s) target the newly identified signaling domain required for iLRP5 signaling and whether additional factor(s) are also critical for endogenous LRP5 function.

In addition to helping elucidate Wnt signaling mechanisms, our initial *in vitro* data suggested that iLRP5 could also be utilized for induction of Wnt signaling *in vivo*, justifying development of the Ubi-Cat mouse model with widespread inducible β-catenin signaling. Ubi-Cat mice should permit analysis of Wnt signaling induction in multiple tissues, consistent with qRT-PCR-based detection of iLRP5 in multiple tissues. The role of Wnt signaling and the pro-neoplastic consequence of Wnt deregulation in mammary and prostate tissues have been well documented [7], [10], [13], [41], [42]. Therefore, we initially selected these two tissue types to examine more carefully the utility of targeted iLRP5 signaling.

In order to demonstrate the cell autonomous activity of iLRP5, we selected prostate B/SCs as our initial working model. Activation of Wnt signaling in prostate progenitors results in the induction of known canonical Wnt target genes, expansion of p63^+^ cell population, enlargement of prostaspheres and development of prostate hyperplasia [13], [30], [31]. Similarly, activation of iLRP5 in LSCs promoted upregulation of known canonical Wnt target genes, such as cyclin D1, c-myc and survivin. Prostasphere assays indicated that iLRP5 activation results in the enlargement of prostaspheres and expansion of the p63^+^ cell population. Moreover, prostate reconstitution assays using Ubi-Cat LSCs revealed that activation of iLRP5 results in expansion of p63^+^ cells and increased cyclin D1 staining. We also observed increased hyperplasia in iLRP5-induced grafts. *In vivo* activation of iLRP5 by administering AP20187 to male Ubi-Cat mice resulted in the progression of prostate tumor development from hyperplasia to adenocarcinoma. Altogether, our *in vitro* and *in vivo* data involving manipulation of Ubi-Cat LSCs and intact prostate, mirrors that of previous reports on the effect of Wnt signaling in prostate progenitors and tumorigenesis [10], [13], [30], [31].

The prostate is a highly organized branched organ that relies on coordinated communication between multiple signaling pathways, such as Wnt, Notch, fibroblast growth factor (FGF) and transforming growth factor β (TGF-β), for proper development. Multiple studies have described the presence of activated canonical Wnt signaling and its importance in prostate architecture organization and cell specification during prostate development and also androgen-dependent regeneration [13], [31], [43]. UGSM-derived tissue provides a suitable niche for glandular development due to its strong inductive characteristic [44], and hence UGSM cells are regularly used for prostate reconstitution assays. Hence, we also tested the effect of iLRP5-mediated Wnt pathway activation within adult prostate stroma and its influence on wildtype LSC cells during prostate reconstitution. Surprisingly, induction of the iLRP5 switch in Ubi-Cat stroma enhanced prostate tissue regeneration when compared to uninduced stromal tissue. This observation emphasizes the importance of Wnt signaling regulation during prostate development. It is likely that iLRP5/Wnt signaling activation induces development-associated signaling pathways that are know to act in a paracrine fashion, such as Notch, FGF and TGF-β, in order to enhance prostate regeneration. Future studies will more closely examine the possible existence of such cross-regulations upon iLRP5 activation.

In this manuscript, we have provided two examples of Ubi-Cat-derived tissues that can be manipulated using a single dimerizing ligand. In addition to the effects of iLRP5 signaling in prostate epithelial and stromal tissue, we also investigated the outcome of iLRP5 induction within mammary cells. Mammary cell transplants into the mammary fat pad also indicated the cell autonomous effects of iLRP5 in mammary epithelial cells during development and homeostasis. In this case, induction of iLRP5 resulted in the formation mammary tumors, demonstrating that induction of unaltered β-catenin is sufficient to drive mammary tumor progression. This result further demonstrates the potency and efficacy of the iLRP5 switch as an inducer of the Wnt signaling pathway. Future studies will investigate the role of canonical Wnt signaling in other tissues in both reconstitution transplant models and in intact Ubi-Cat mice. Additional animal models based on tissue-specific regulatory elements or conditional, tissue-specific iLRP5 expression should further broaden the utility of this technology.

### Conclusions

Overall, this study has demonstrated the generation of a novel inducible Wnt signaling switch by manipulating the Wnt pathway co-receptor LRP5. We observed that membrane recruitment of iLRP5 via CID leads to activation of the Wnt signaling pathway. Oligomerization of LRP5 is also demonstrated to be a potent inducer of Wnt pathway induction, independent of membrane recruitment. This observation led us to generate an inducible Wnt switch (iLRP5) using only the cytoplasmic domain of LRP5. The activation of iLRP5 is achieved via CID technology, in which iLRP5-mediated Wnt signaling induction is dependent on exposure to synthetic homodimerizer drugs, like AP20187.

Next, we investigated the molecular and biochemical events that follow upon iLRP5 activation, leading to Wnt signaling activation. We were able to demonstrate that iLRP5 interacts with Dvl; however, this interaction is not critical for the downstream activity of iLRP5. Instead, the iLRP5 switch has the ability to induce Wnt signaling by sequestering the Wnt inhibitory molecule, Axin. The iLRP5/Axin interaction appears sufficient to activate the Wnt pathway, even in the absences of full-length Dvl, suggesting that iLRP5 does not require Dvl activity or membrane localization. We also showed that iLRP5 phosphorylation via CK1 is required for proper iLRP5 activity. Finally, we generated the Ubi-Cat transgenic mouse model. In two distinct organ systems, Ubi-Cat mice demonstrated their utility as a practical tool for investigating the effect of Wnt signaling induction *in vitro* and *in vivo*.

## Materials and Methods

### Animals, cells, reagents, and constructs

All mice were housed at the Baylor College of Medicine (BCM) Transgenic Mouse Facility in accordance with the guidelines of the Institutional Animal Care and Use Committee (IACUC) for BCM. This study was IACUC reviewed and approved within protocol #AN-1428. HEK293 (ATCC, Manassas, VA) cells were maintained in DMEM, 10% FCS and antibiotics. Rapalog-B/AP22783 (Rap-B), FK1012 and AP20187 were provided by Ariad Pharmaceuticals (Ariad Pharmaceuticals, Cambridge, MA). Rabbit anti-myc antibody (Covance/CRP), mouse anti-hemagglutinin (HA) epitope (HA-11, Covance/CRP) and mouse anti-myc antibody (9B11, Cell Signaling Technology, Danvers, MA) were used for immunoprecipitation and Western blotting. A phospho-specific anti-PPPS_P_P antibody was kindly provided by C Niehr [24]. Cholera toxin B (CTx-B-TRITC) was used to detect glycosphingolipid in membrane lipid-rafts (List Biological Laboratories, Campbell, CA). The PI3-Kinase inhibitors wortmannin (W), ERK inhibitor PD98059 (PD), Akt inhibitor (Akt-i) and casein kinase inhibitor (CK1-I) were purchased from Calbiochem/EMD Chemicals (San Diego, CA).

To generate TOP-SEAP and FOP-SEAP reporter plasmids, the LEF-responsive promoter (“TOP”) and mutant LEF-unresponsive promoter (“FOP”) from TOPflash or FOPflash (Upstate Biotechnology/Millipore, Billerica, MA) were PCR-amplified using N-terminal *Sal*I-, and C-terminal *Not*I-linkered primers and subcloned into *Xho*I/*Not*I-digested pWG018-SEAP. Axin-myc and Dvl constructs were kindly provided by H Varmus (MSKCC). Full-length human LRP5 and LRP6 constructs, pCDNA6-hLRP5 and pCDNA6-hLRP6, were kindly provided by P McCrea (MDACC). The construction of Fyn-myristoylated FRB_l_2 (pBJ5/M_F_-FRB_l_2-E) was previously described [21]. For the construction of M_F_-LRP5c, the full-length cytoplasmic domain of LRP5c (LRP5_1408–16165_) was PCR-amplified using N-terminal *Xho*I-, and C-terminal *Sal*I-linkered primers and subcloned in-frame into *Xho*I-*Sal*I digested pBJ5/M_F_-FRB_l_2-E.

To generate pSH1-F_PK_3-LRP5c (F3-LRP5c), pSH1-F_PK_3-GFP-LRP5c (F3-GFP-LRP5c) and pSH1-F_v′_-F_vls_-LRP5c (iLRP5), PCR-amplified LRP5c was subcloned into *Sal*I-digested vectors pSH1-F_PK_3, pSH1-F_PK_3-GFP or pSH1-F_v′_-F_vls_. All truncated LRP5c mutants; iLRP5Δ1 (LRP5_1456–1616_), iLRP5Δ2 (LRP5_1490–1616_), iLRP5Δ3 (LRP5_1515–1616_), iLRP5Δ4 (LRP5_1532–1616_) and iLRP5Δ5 (LRP5_1553–1616_) were PCR-amplified using primers containing N-terminal *Xho*I, and C-terminal *Sal*I linkers and subcloned into the *Sal*I site of pSH1-F_v′_-F_vls_-E or the *Sal*I site 3′ of GFP in pSH1-F_PK_3-GFP-E [20].

### TCF-SEAP and NF-κB-SEAP (secreted alkaline phosphatase) reporter assays

SEAP reporter assays in HEK293 cells were carried out in 6- or 12-well plates as described previously with minor modifications [21]. Briefly, HEK293 cells in 6-well plates were transfected with 1 µg of indicated plasmids along with TOP-SEAP reporter plasmid (0.5–1 µg/well), FOP-SEAP or NF-κB-SEAP reporter plasmid as a negative control using FuGene 6 reagent (Roche). The following day, cells were washed and collected with 5 ml of complete media and 200 µl aliquots were replated in triplicate in 96-well plates with indicated concentrations of CIDs or 50 ng/ml of recombinant hCD40 ligand (R&D system, Minneapolis, MN) and further incubated for ∼24 h. For the inhibitor study, HEK293 cells in 24-well plates were transfected with indicated plasmids and were incubated with indicated concentration of each inhibitor for 24 h. The following day, cells were washed and further incubated with indicated concentrations of CIDs for ∼24 h. Thereafter, supernatants were collected and analyzed for SEAP activity. Briefly, saran wrapped plates were heat-inactivated (HI) at 68°C for 45′. 100 µL aliquots of HI supernatants were mixed with 100 µL of 2 mM 4-methylumbilliferyl phosphate (Sigma) in 2 M diethanolamine (Sigma) and fluorescence (Ex:355, Em: 460 nm) was analyzed at various intervals. For each experiment, we calculated the average of replicates and subtracted background signals for non-transfected cell supernatants with substrate.

### Construction of transgene

The H2K-iLRP-I-CBR (Ubi-Cat) construct was generated in multiple cloning steps. First, an *Eco*RV-*Not*I (blunt) 2-kb fragment from H2K-I-LTR (kind gift of Dr. Jos Domen, Medical College of Wisconsin) was cloned into *Bam*HI-digested (and blunted) KBPA vector to generate H2K-KBPA. Next, the 1.3-kb *Not*I-*Eco*RI digested iLRP5 fragment was removed from the pSH1-S-F_v′_F_vls_-LRP5-FLAGx2 construct. This fragment was cloned into *Not*I/*Mun*I-digested pSH1(Mn)-IRES-Lip (N,E) to get pSH1/iLRP5-IRES. To obtain the reporter, we PCR-amplified the click beetle red-shifted luciferase (CBR) reporter using primers, 5CBRX: 5′-caagtcctcgagGTAAAGCGTGAGAAAAATGTCATC-3′ and 3CBRE: 5′-gacttgaattcTTACTAACCGCCGGCCTTCACC-3′ to get the 1664-bp CBR (X,E) fragment. The amplified product was digested via *Xho*I and *Eco*RI and the fragment was cloned into XhoI (partial digest)/EcoRI-digested pSH1/iLRP5-IRES to obtain pSH1/iLRP5-IRES-CBR. The NotI site was converted to the *Eco*RI site using primers Not2Eco5: 5′-ggccgcaagaattcaatc-3′ and Not2Eco3: 5′-ggccgattgaattcttgc-3′ to get pSH1(Nt)/iLRP5-IRES-CBR. This 3.6-kb fragment was then cloned into H2K-KBPA to get H2K/iLRP5-IRES-CBR. Transgene fragment was isolated by *Spe*I restriction digest. The fragment was gel purified using Qiagen gel purification kit (Qiagen, Valencia, CA). The purified fragments were used for injection into FVB strain-derived embryonic stem cells.

### *In vitro* sphere cultures, sphere collection, RNA isolation, qRT-PCR analysis and preparation of prostaspheres for immunostaining

Ubi-Cat LSCs were cultured in Matrigel (BD Bioscience, Franklin Lakes, NJ) as previously described [29]. LSCs were cultured in prostate epithelial growth medium (PrEGM) (Lonza, Walkersville, MD) or PrEGM supplemented with 100 nM AP20187(Ariad Pharmaceuticals, Cambridge, MA). The media was refreshed every two days. In order to isolate Matrigel-embedded prostaspheres for gene expression or immunostaining analysis, the cultures were treated with dispase (Stem Cell Technologies, Vancouver, BC) for 45 min at 37°C. Spheres were washed with cold PBS prior to subsequent analysis. RNA isolation was performed using TRIzol (Invitrogen, Carlsbad, CA) according to the manufacturer's instructions. The cDNA synthesis was performed using qScript™ cDNA Synthesis Kit (Quanta Biosciences, Gaithersburg, MD), and qRT-PCR analysis was performed using TaqMan® Gene Expression Assay protocols using an ABI Prism 7000 sequence detection system (Applied Biosystems, Foster City, CA). For Immunostaining analysis, isolated spheres where placed in histoGel (Lab Storage System Inc., St. Peters, MO) and fixed for 10 min in 10% neutral phosphate-buffered formalin then washed with 70% EtOH. HistoGel “plugs” were embedded in paraffin and sectioned for immunostaining analysis.

### Prostate reconstitution assays

For *in vivo* reconstitution assays LSCs from 6–8 week-old adult WT or Ubi-Cat mice were mixed with adult prostate stroma or E-18 Rat UGSM. UGSM cells were prepared as described [29]. Ubi-Cat LSCs (1×10^5^) and 1×10^5^ UGSM or 1×10^5^ WT LSCs and 1×10^5^ adult prostate stroma were mixed and pelleted by centrifugation at 600 RCF. Pelleted cells were resuspended in 100 µl of Matrigel™ (BD Bioscience) on ice. The Matrigel:Cell mixture was injected subcutaneously into CD1^nu/nu^ mice. Mice were treated with i.p. injection of AP20187 (Ariad Pharmaceuticals) at 2 mg/kg in drug diluent (16.7% propanediol, 22.5% PEG400, 1.25% TWEEN 80) or drug diluent alone twice a week for 8 weeks. The grafts were isolated for further analysis.

### Immunofluorescence (IF), Immunohistochemistry (IHC)

Formalin-fixed samples were embedded in paraffin and 5-µm sections were deparaffinized and hydrated in 100% and 95% EtOH steps and rinsed in water. Hydrated sections were stained with hematoxylin and eosin (H&E). Prior to IF and IHC, Sodium citrate (10 mM) antigen retrieval was performed, and sections were blocked with goat serum or M.O.M (Vector Laboratories, Inc. Burlingame, CA) kit for a minimum of 1 h at RT. When performing IHC, cellular peroxidases were neutralized using 3% H_2_O_2_ for 10 min. Sections were incubated with 1° antibody ON at 4°C. Primary antibody dilutions were as follows: β-catenin 1∶500 (Cell Signaling, Danvers, MA), p63 1∶500 (Thermo Fisher Scientific, Fremont, CA), cyclin D1 (SP4) (Thermo Fisher Scientific, Fremont, CA). Secondary antibody incubation was performed for 1 h at RT. Secondary antibodies (Invitrogen) were donkey anti-mouse Alexa Fluor 599, goat anti-rabbit Alexa Fluor 488. the slides were overlaid with ProLong® Gold anti-fade reagent with DAPI (Invitrogen).

For IHC, slides were incubated with secondary biotinylated goat anti-rabbit (1∶500 for 1 h at RT). Staining was performed by treatment with ABC reagent for 45 min followed by DAB for 3 min (Vector Laboratories Inc., Burlingame, CA) and counterstained with hematoxylin.

### Site-Directed Mutagenesis

The iLRP5 mutants were generated by site-directed mutagenesis using the QuikChange Site-Directed Mutagenesis Kit (Stratagene, La Jolla, CA). Oligonucleotides for different iLRP5 mutants were designed according to the manufacturer's protocol: iLRP5-YSS→FAA, 5′-GTACAACATGGACATGTTCT**T**C**G**CT**G**CAAACATTCCGGCCACTG CGAG-3′; iLRP5-YRPY→FLPF, 5′-CCGGCCACTGCGAGACCGT**T**C**TT**GCCCT**T**CATCA TTCGA GGA ATGGCG-3′. (Bold characters denote mutated nucleotides.)

### iLRP5 Cross-Linking

HEK293 cells were transfected with 1.5 µg of iLRP5 via FuGene 6 reagent (Roche) in 12-well format. 24-hr post-transfection, iLRP5 was activated via 100 nM/ml of AP20187 for an additional 24-hr. Prior to cell lysis on the following day, fresh AP20187 was added for an additional 2-hr. Cells were lysed in 50 µL of mild detergent (20 mM MOPS, pH 7.2, 2 mM EGTA, 10 mM MgCl_2_, 1% Triton X-100). Cell lysates were crosslinked using 10 mM of disuccinimidyl glutarate (Thermo Scientific, Rockford, IL) as suggested by the manufacturer. Samples were analyzed via Western Blot analysis.

### Statistical analysis

Statistical significance was determined based on the student *t*-test between indicated groups.

## Supporting Information

Figure S1 **M_F_-LRP5/6 constitutively induces TCF/β-catenin activity.** (**A**) LiCl_2_-dependent, specific induction of TCF4/β-catenin-responsive reporter, TCF-SEAP, activity. 293 cells were transiently transfected with 1 µg of TOP-SEAP, FOP-SEAP reporter construct, or vector control. TOP-SEAP reporter activity was measured 24 hours after administration of indicated amounts of LiCl_2_. (**B**) Constitutive β-catenin induction by lipid raft targeting of the intact intracellular domains of LRP5 and LRP6 (M_F_-LRP5c and M_F_-LRP6c) but not by the triple PPPSP domain. 293 cells were transiently transfected with 1 µg of myristoylated EGFP (M_F_-EGFP), LRP5c (M_F_-LRP5c), LRP6c (M_F_-LRP6c), or the triple PPPSP domain of LRP5 (M_F_-LRP5-3xPPPSP) along with 1 µg of TOP-SEAP or FOP-SEAP reporter constructs. Twenty-four hours after transfection, SEAP reporter activity was measured. Data is representative of at least three independent experiments.
(TIF)

Figure S2 **Effect of Axin on M_F_-LRP5c-mediated β-catenin activation.** 293 cells were transiently transfected with 1 µg of TCF-SEAP reporter and 1 µg of M_F_-GFP (-) or M_F_-LRP5c expression vector along with indicated amounts (µg) of myc-tagged Axin. TCF-SEAP activity was measured 24-hours after transfection. The amount of protein expression was shown by anti-Myc and anti-HA blotting (bottom). Data is representative of at least three independent experiments.
(TIF)

Figure S3 **Western blot analysis examining the expression of iLRP5/DIX, iLRP5/Axin and iLRP5/Dvl co-transfection experiments.** 293 cells were transfected with 1 µg TCF-SEAP reporter along with 0.5 µg of iLRP5 and the indicated amounts (µg) of dominant negative disheveled-FLAG (**A**) or Axin-Myc constructs (**B**) or 0.5 µg disheveled-FLAG construct (**C**). Twenty-four hours after transfection, 100 nM CID was added to the cells and SEAP activity was measured following additional 24 h incubation. Cell lysate was obtained for Western blot analysis. Actin probe was used to assess the overall protein load in each lane.
(TIF)

Figure S4 (**A, B**)**. Constitutive phosphorylation of PPPSP sites in iLRP5 mutants with or without dimerization.** 293 cells were transfected with 1 µg of indicated mutants of iLRP5 along with 1 µg of the Axin expression construct. After 24-hour treatment with 100 nM AP20187 (+CID), whole cell lysates were immunoprecipitated with mouse anti-HA antibody (A, bottom) or rabbit anti-Myc antibody (B, middle). Phosphorylation of PPPSP sites in iLRP5 mutants (A, top) and co-immunoprecipitation of LRP5 mutants with Axin (B, top) were analyzed by anti-pS1490 (A, α-pS1490) and anti-HA blot (B, α-HA). Axin was immunoprecipitated from whole cell lysates with anti-Myc antibody (α-Myc). Co-immunoprecipitation of LRP5c was analyzed by anti-HA blot (α-HA). The amount of immunoprecipitated iLRP5 mutants (A, rabbit α-HA) and iLRP5 mutant expression (B) was determined by HA blot (B, Input: α-HA).
(TIF)

Figure S5 **iLRP5 promotes nuclear localization of β-catenin in prostate epithelial cells.** Cellular localization of β-catenin in prostate tissue was analyzed via immunostaning. CID mediated iLRP5 dimerization results in activation of canonical Wnt singanling as suggested by presence of nuclear β-catenin (arrow heads).
(TIF)

Figure S6 **iLRP5 promotes prostate hyperplasia.** Activation of iLRP5 in Ubi-Cat mice for 36–44 weeks promotes proliferative phenotype within epithelial and stromal compartment. The cellular proliferation is more prominent in the ventral and dorsal lobes. Ventral and dorsal lobes demonstrate epithelial proliferation and significant stromal thickening. Bar = 200 µm.
(TIF)
