# Supplementary figures and images for: Activation of Wnt Signaling by Chemically Induced Dimerization of LRP5 Disrupts Cellular Homeostasis
Source: PLoS One. 2012 Jan 27;7(1):e30814. doi: 10.1371/journal.pone.0030814 (PMC3267738; doi:10.1371/journal.pone.0030814)

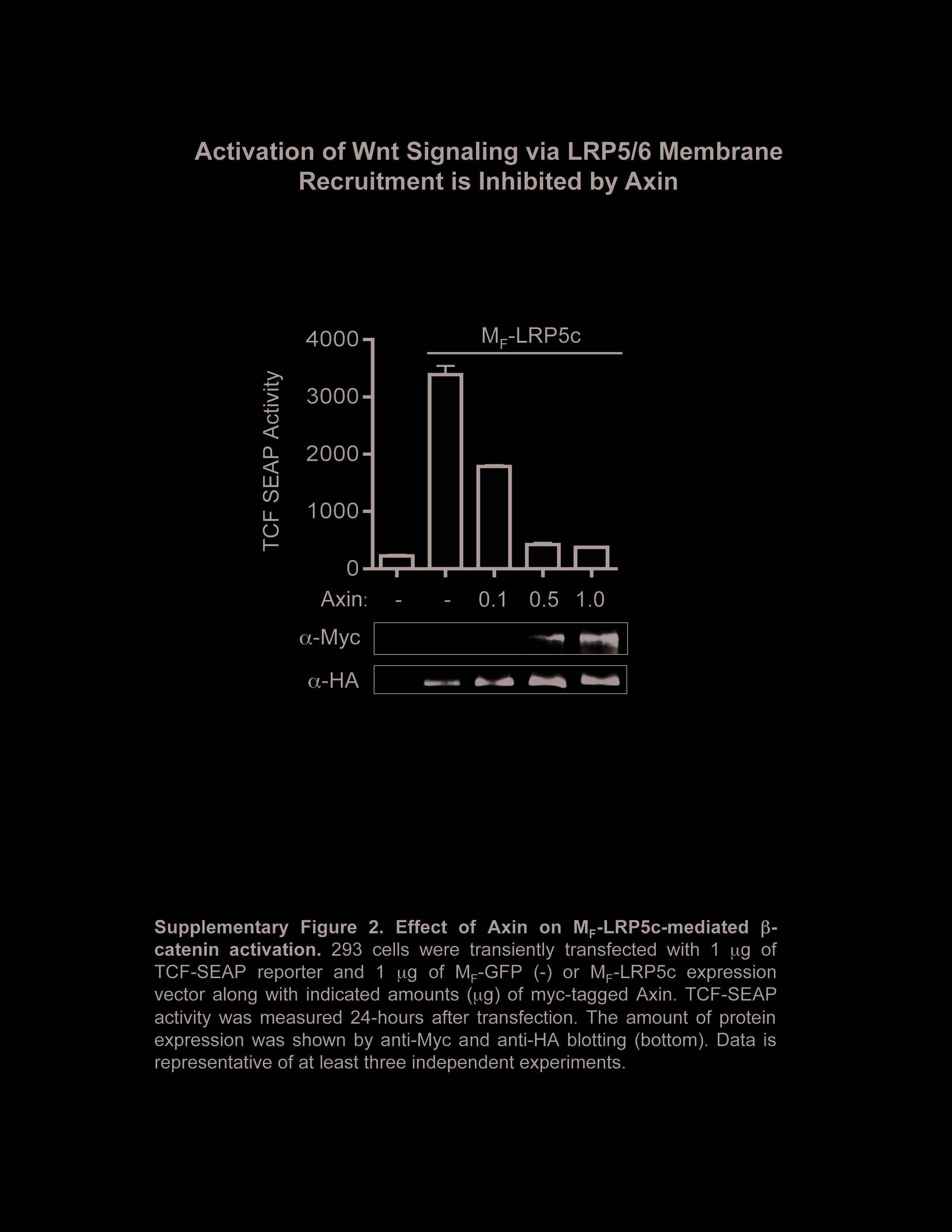

Supplement: Figure S2 — Effect of Axin on MF-LRP5c-mediated β-catenin activation. 293 cells were transiently transfected with 1 µg of TCF-SEAP reporter and 1 µg of MF-GFP (-) or MF-LRP5c expression vector along with indicated amounts (µg) of myc-tagged Axin. TCF-SEAP activity was measured 24-hours after transfection. The amount of protein expression was shown by anti-Myc and anti-HA blotting (bottom). Data is representative of at least three independent experiments. (TIF) [file pone.0030814.s002.tif]

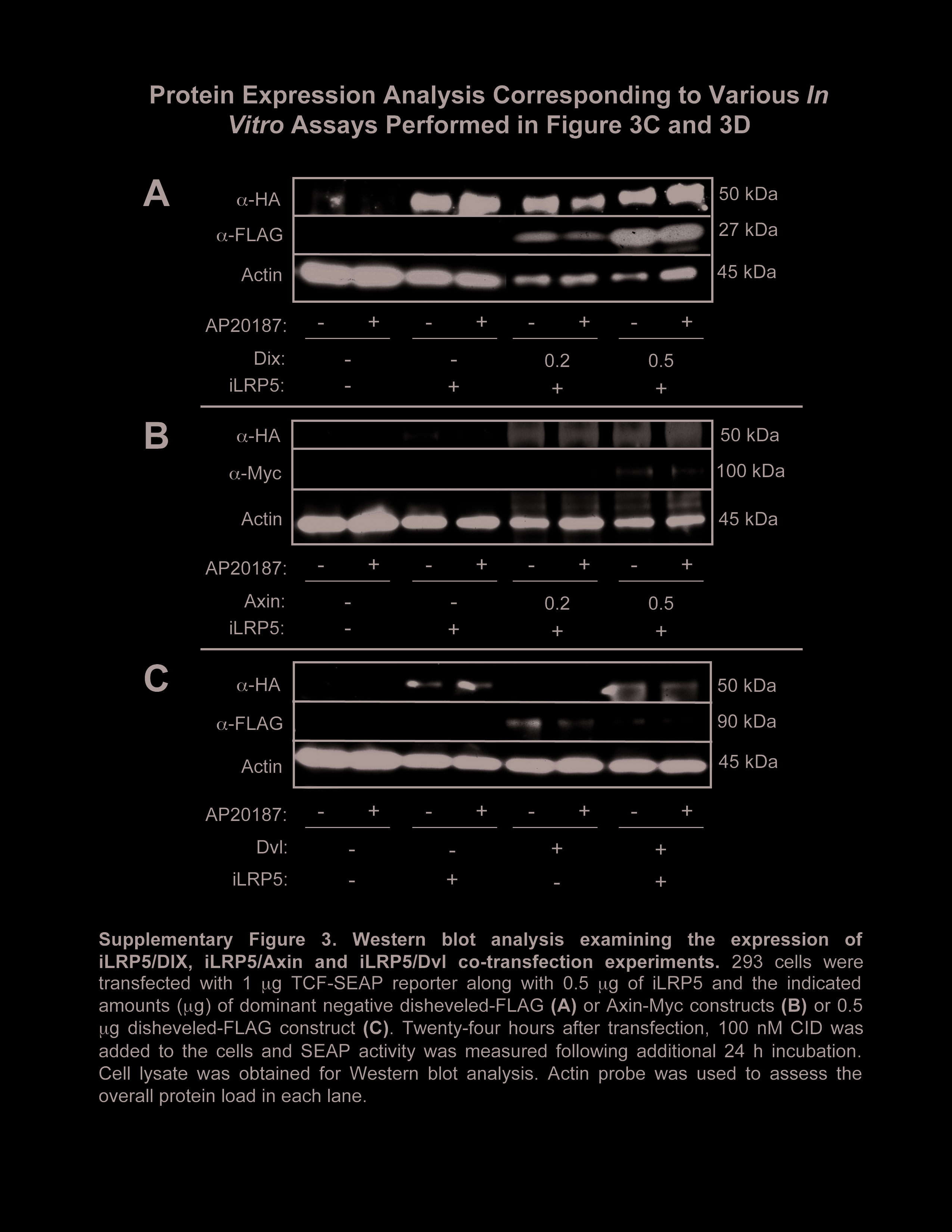

Supplement: Figure S3 — Western blot analysis examining the expression of iLRP5/DIX, iLRP5/Axin and iLRP5/Dvl co-transfection experiments. 293 cells were transfected with 1 µg TCF-SEAP reporter along with 0.5 µg of iLRP5 and the indicated amounts (µg) of dominant negative disheveled-FLAG (A) or Axin-Myc constructs (B) or 0.5 µg disheveled-FLAG construct (C). Twenty-four hours after transfection, 100 nM CID was added to the cells and SEAP activity was measured following additional 24 h incubation. Cell lysate was obtained for Western blot analysis. Actin probe was used to assess the overall protein load in each lane. (TIF) [file pone.0030814.s003.tif]

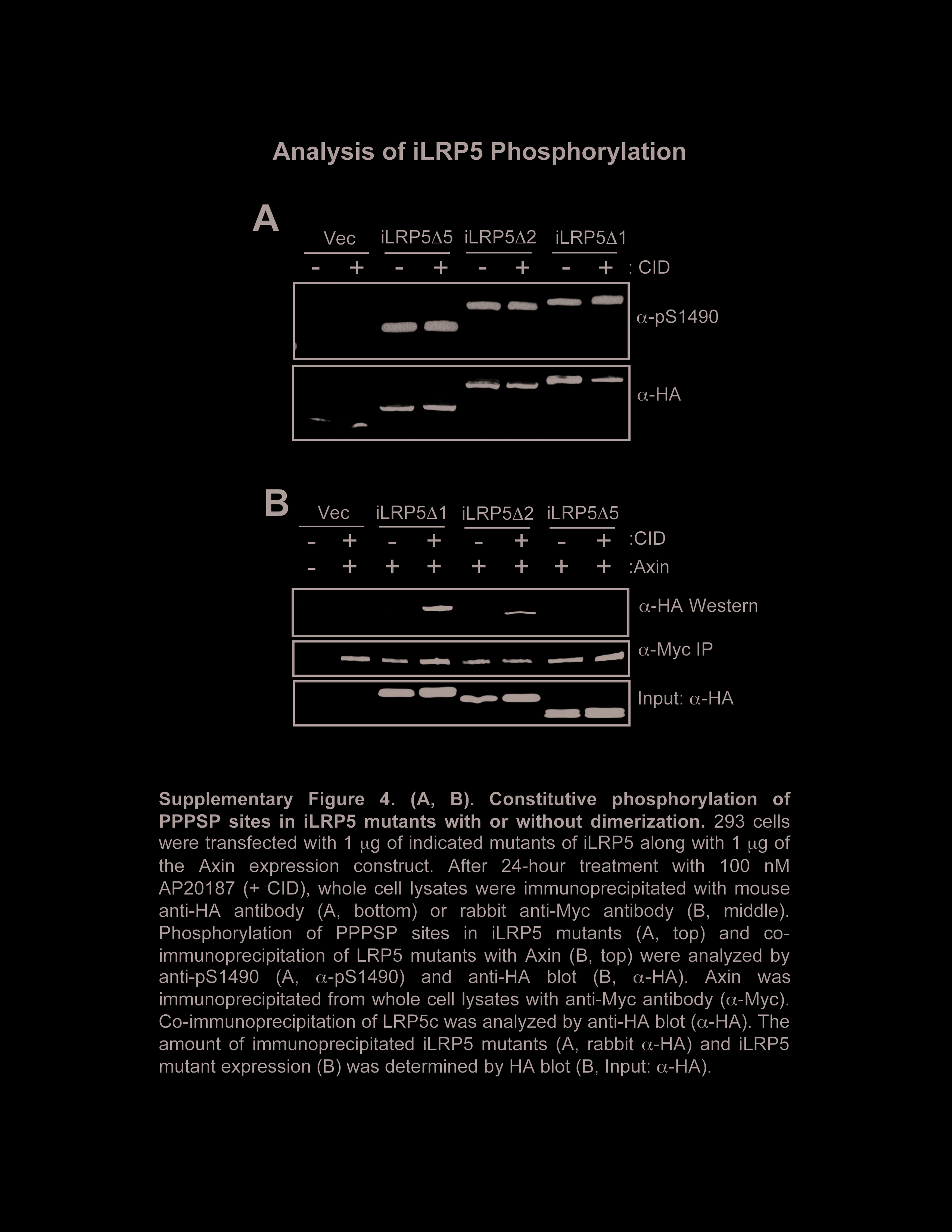

Supplement: Figure S4 — (A, B). Constitutive phosphorylation of PPPSP sites in iLRP5 mutants with or without dimerization. 293 cells were transfected with 1 µg of indicated mutants of iLRP5 along with 1 µg of the Axin expression construct. After 24-hour treatment with 100 nM AP20187 (+CID), whole cell lysates were immunoprecipitated with mouse anti-HA antibody (A, bottom) or rabbit anti-Myc antibody (B, middle). Phosphorylation of PPPSP sites in iLRP5 mutants (A, top) and co-immunoprecipitation of LRP5 mutants with Axin (B, top) were analyzed by anti-pS1490 (A, α-pS1490) and anti-HA blot (B, α-HA). Axin was immunoprecipitated from whole cell lysates with anti-Myc antibody (α-Myc). Co-immunoprecipitation of LRP5c was analyzed by anti-HA blot (α-HA). The amount of immunoprecipitated iLRP5 mutants (A, rabbit α-HA) and iLRP5 mutant expression (B) was determined by HA blot (B, Input: α-HA). (TIF) [file pone.0030814.s004.tif]

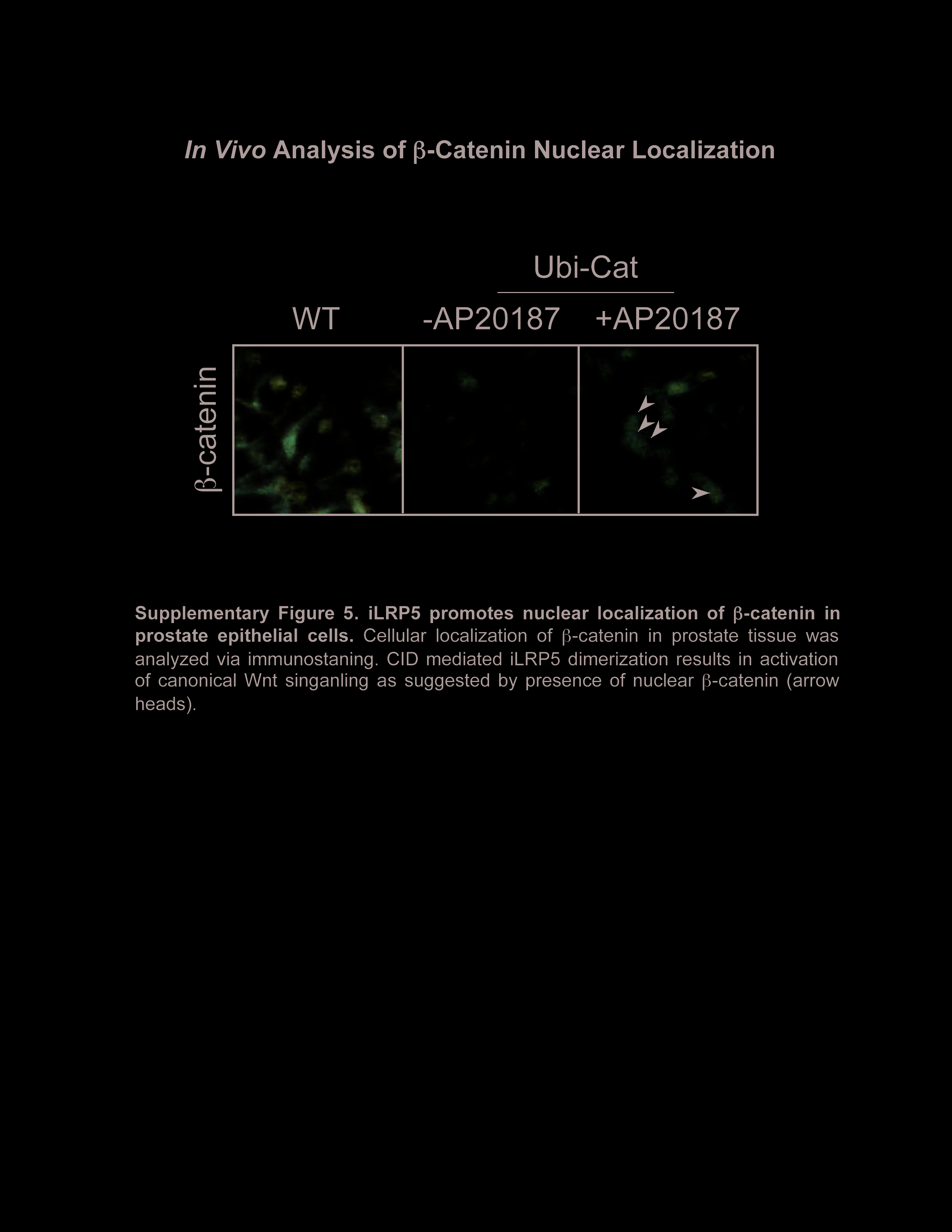

Supplement: Figure S5 — iLRP5 promotes nuclear localization of β-catenin in prostate epithelial cells. Cellular localization of β-catenin in prostate tissue was analyzed via immunostaning. CID mediated iLRP5 dimerization results in activation of canonical Wnt singanling as suggested by presence of nuclear β-catenin (arrow heads). (TIF) [file pone.0030814.s005.tif]

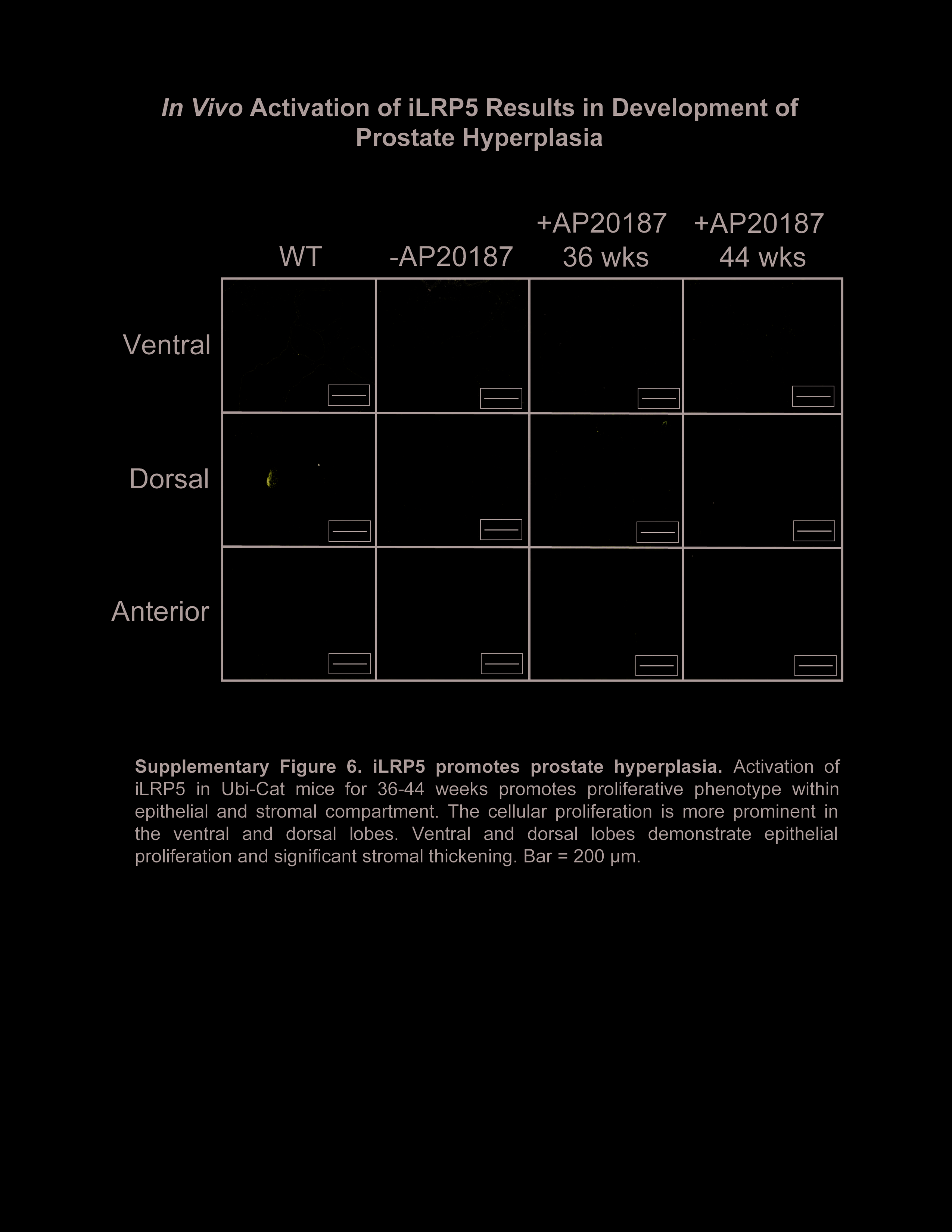

Supplement: Figure S6 — iLRP5 promotes prostate hyperplasia. Activation of iLRP5 in Ubi-Cat mice for 36–44 weeks promotes proliferative phenotype within epithelial and stromal compartment. The cellular proliferation is more prominent in the ventral and dorsal lobes. Ventral and dorsal lobes demonstrate epithelial proliferation and significant stromal thickening. Bar = 200 µm. (TIF) [file pone.0030814.s006.tif]
